# Therapeutic faecal microbiota transplantation controls intestinal inflammation through IL10 secretion by immune cells

**DOI:** 10.1038/s41467-018-07359-8

**Published:** 2018-12-05

**Authors:** Claudia Burrello, Federica Garavaglia, Fulvia Milena Cribiù, Giulia Ercoli, Gianluca Lopez, Jacopo Troisi, Angelo Colucci, Silvia Guglietta, Sara Carloni, Simone Guglielmetti, Valentina Taverniti, Giulia Nizzoli, Silvano Bosari, Flavio Caprioli, Maria Rescigno, Federica Facciotti

**Affiliations:** 1 Department of Experimental Oncology, European Institute of Oncology IRCCS, via Adamello 16, Milan, 20139 Italy; 2 Department of Oncology and Hemato-oncology, Università degli Studi di Milano, via F. Sforza 28, Milan, 20122 Italy; 3 Pathology Unit, Fondazione IRCCS Cà Granda, Ospedale Maggiore Policlinico, via F. Sforza 35, Milan, 20135 Italy; 4 Department of Medicine, Surgery and Dentistry, “Scuola Medica Salernitana”, University of Salerno, Baronissi, 84081 SA Italy; 5 Theoreo srl, Spin-off company of the University of Salerno, Via degli Ulivi 3, 84090 Montecorvino Pugliano, SA Italy; 6 European Biomedical Research Institute of Salerno (EBRIS), Via S. de Renzi, 3, 84125 Salerno, SA Italy; 7 Laboratory of Mucosal Immunology and Microbiota, Humanitas Clinical and Research Center, Via Manzoni 56, Milan, 20089 Italy; 8 Department of Food Environmental and Nutritional Sciences (DeFENS), Università degli Studi di Milano, Milan, 20133 via Celoria 2, Italy; 9 Gastroenterology and Endoscopy Unit, Fondazione IRCCS Cà Granda, Ospedale Maggiore Policlinico, via F. Sforza 35, Milan, 20135 Italy; 10 Department of Pathophysiology and Transplantation, Università degli Studi di Milano, via F. Sforza 28, Milan, 20135 Italy

## Abstract

Alteration of the gut microbiota has been associated with different gastrointestinal disorders. Normobiosis restoration by faecal microbiota transplantation (FMT) is considered a promising therapeutic approach, even if the mechanisms underlying its efficacy are at present largely unknown. Here we sought to elucidate the functional effects of therapeutic FMT administration during experimental colitis on innate and adaptive immune responses in the intestinal mucosa. We show that therapeutic FMT reduces colonic inflammation and initiates the restoration of intestinal homeostasis through the simultaneous activation of different immune-mediated pathways, ultimately leading to IL-10 production by innate and adaptive immune cells, including CD4^+^ T cells, iNKT cells and Antigen Presenting Cells (APC), and reduces the ability of dendritic cells, monocytes and macrophages to present MHCII-dependent bacterial antigens to colonic T cells. These results demonstrate the capability of FMT to therapeutically control intestinal experimental colitis and poses FMT as a valuable therapeutic option in immune-related pathologies.

## Abstract (web summary)

Faecal microbiota transplantation (FMT) is becoming a therapeutic option in several gastrointestinal disorders. Here, Burrello et al. study the immunological mechanisms by which FMT reduces colonic inflammation and initiates the restoration of intestinal homeostasis in a mouse model of colitis.

## Introduction

The gut mucosa constitutes a unique environment exposed to more than 10^14^ commensal bacteria, which establish a mutualistic relationship with the host, providing metabolic functions and contributing to shape the immune system^1^. Maintenance of intestinal homeostasis requires several methods to physically confine commensal bacteria to the intestinal lumen, while keeping the full capability to control colonization by pathogenic bacteria^1^. Variations of this equilibrium induce the recruitment and expansion of several immune cell types contributing to initiate and propagate intestinal inflammation, or to restore homeostasis by activating tolerogenic mechanisms^2^.

Alteration in the composition of the gut microbiota (dysbiosis) has been associated with a wide range of gastrointestinal diseases, including recurrent *C. difficile* infection (CDI)^3^, inflammatory bowel diseases (IBD, Crohn’s disease, CD, and Ulcerative colitis, UC)^4,5^ and colorectal cancer (CRC)^6^.

Current theories suggest that IBD onset is secondary to an exaggerated reaction of gut-associated lymphoid tissue against the luminal microbiota^7^. Whether this is a primary defect or it is secondary to intestinal dysbiosis is still debated. Indeed, a reduced biodiversity in both mucus-associated and faecal bacterial communities has been observed both in IBD patients and in their first degree relatives^4,8,9^. Moreover, IBD patients showed reduced diversity of their gut microbiome, expansion of pro-inflammatory bacteria like *Enterobacteriaceae* and *Fusobacteriaceae* and depletion of phyla with anti-inflammatory functional properties such as *Firmicutes*^10^. Additionally, changes in mucus-associated microbiota have been observed in the ileal mucosa of children with treatment-naïve IBD, independently from intestinal inflammation^4^. These observations suggest that intestinal dysbiosis may be causally related and might precede mucosal inflammation in IBD patients. This has also been confirmed by the induction of experimental inflammation upon transfer of dysbiotic microbiota or single commensal bacterial species in germ-free mice^11^.

Intestinal epithelial damage has been proposed to be a key event favouring bacteria translocation out of the intestinal lumen, thus facilitating the recognition of antigens derived from the dysbiotic microflora by pathogenic T cells in IBD patients^7^. A complex physical barrier composed by a monolayer of polarized epithelial cells has been developed to avoid exposure to the intestinal microbiome^12^. Additionally, to prevent the bacterial access to epithelial cells, mucous layers of different densities line on top of the epithelium. Other host’s cellular components contribute to homeostasis maintenance through the secretion of antimicrobial peptides (AMPs)^12^.

Manipulation of the intestinal microbiome is becoming a therapeutic option in several gastrointestinal disorders^13^. Faecal microbiota transplantation (FMT), i.e. the infusion of healthy donor faeces in the gut of a recipient to treat a disorder associated with microbiota alterations, is receiving great consideration thanks to its effectiveness against refractory and recurrent CDI^14,15^. Increasing evidences from recent Randomized Clinical Trials (RCTs) are also supporting the possibility to utilise FMT to treat mild-to-moderate UC patients^16–18^. Despite its recent successes, it is still largely unknown how FMT functionally modulates the intestinal immune system. Very limited data are available on the therapeutic re-equilibration of the gut microbiota in murine models of intestinal inflammation^19,20^.

Since the intestinal immune system is sensitive to variations of luminal and mucus-associated bacteria, we postulated that therapeutic FMT administered during intestinal inflammation might directly modulate both innate and adaptive mucosal immune responses towards the control of intestinal inflammation. Given the predominant role of CD4^+^ T cells in orchestrating IBD-related inflammatory processes^21^, largely due to their differentiation into IFNγ/IL-17-secreting effector subtypes, we focused our primary analysis on the functional variations occurring in these cell populations upon FMT. The effect of microbiota alterations during colitis induction and upon FMT treatment in colitogenic mice was additionally assessed on iNKT cells, a subset of αβ-T cells recognizing both self- and microbial-derived glycolipids^22,23^ and heavily influenced by bacterial-derived antigens in early life^24,25^. We also recently showed that colonic iNKT cells are sensitive to microbiota alterations occurring after short term antibiotic treatment^26^. To selectively track this rare cell subset, we took advantage of a novel reporter mouse strain, the CXCR6^EGFP^ mouse^27^.

Here we demonstrate that therapeutic administration of FMT during experimental colitis exerts beneficial effects, which are associated to the simultaneous activation of several anti-inflammatory pathways. Upon FMT, variations in the intestinal ecology towards IL-10-inducing microbial communities and the production of tolerogenic IL-10 by mucosal innate and adaptive cell subsets altogether concur to resolve the inflammation.

## Results

### Therapeutic FMT reduces inflammation in DSS-induced colitis

CXCR6-^EGFP/+^ mice^27^ are susceptible to DSS-induced colitis (Supplementary Fig. 1) and can be used to track intestinal CD4^+^ T cell subsets, including the relatively rare iNKT cells (Supplementary Fig. 2), under homeostatic conditions^26^ and during experimental intestinal inflammation.

**Fig. 1:**
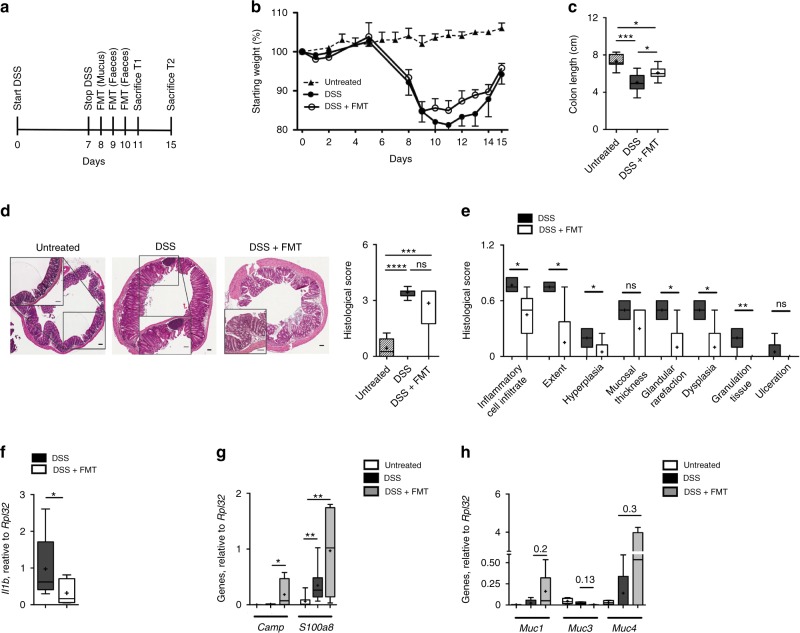
Therapeutic FMT ameliorates DSS-induced experimental colitis. **a** Schematic representation of FMT treatment during acute DSS experimental colitis. **b**, **c** Weight loss (**b**) and colon length (**c**) of untreated (triangles and striped boxes), colitic (black dots and black boxes), or colitic mice treated with FMT (white dots and white boxes). **d**, **e** H&E staining (scalebar 100 µm) and cumulative histological score of colon specimens obtained from DSS-treated and FMT-treated mice; (**e**) Detailed histological evaluation of mice with decreased histological score (white boxes) compared to DSS-treated mice (black boxes). **f** Colonic expression levels of *Il1β* in colitic (black boxes) and FMT-treated (white bars) mice. **g**, **h** Colonic expression levels of *Camp*, *S100A8* (**g**) and *Muc1, Muc3, Muc4* (**h**) in untreated (white boxes), DSS treated (black boxes) or DSS + FMT-treated (gray boxes) mice. Statistical significance was calculated using a Mann–Whitney test for comparison within two groups or Kruskal–Wallis test with Dunn’s multiple comparison correction within more than two groups. *P < 0.05, **P < 0.01, ***P < 0.001, ****P < 0.0001 were regarded as statistically significant. Outliers were detected with Grubb’s test. Non parametric distributions were represented as median + /- interquartile range. In Box and whiskers plots, centre line represents median; cross, represents mean. In (**b**) UT *n* = 10, DSS *n* = 10, DSS + FMT *n* = 10. In (**c**) UT *n* = 12, DSS *n* = 12, DSS + FMT *n* = 14. In (**d**) UT *n* = 8, DSS *n* = 15, DSS + FMT *n* = 19. In (**f**–**h**) UT *n* = 5, DSS *n* = 6, DSS + FMT *n* = 7

Upon establishment of DSS-induced acute colitis, the functional effects of therapeutic FMT were evaluated. Mucus and faeces derived from normobiotic mice were collected and microbiota transfer was performed in colitic mice by oral gavage for 3 consecutive days according to the scheme described in Fig. 1a. At sacrifice, FMT-treated mice showed reduced signs of intestinal inflammation, as indicated by decreased weight loss (Fig. 1b) and increased colonic length (Fig. 1c and Supplementary Fig. 3a). These effects were associated to an amelioration of the intestinal inflammation measured by the histological score upon FMT (Fig. 1d, e and Supplementary Fig. 3b). Moreover, in agreement with previously published reports^19^, FMT reduced the expression of the pro-inflammatory *Il1β* (Fig. 1f and Supplementary Fig. 3c) in the colonic mucosa.

To evaluate whether FMT might exert protective effects on gut barrier functions, the colonic expression of antimicrobial peptides and mucins was also tested (Fig. 1g, h). The expression of the tight junction protein Zo-1 was not affected upon FMT treatment in the colons of colitic mice (Supplementary Fig. 3d). On the contrary, Camp and S100A8, two antimicrobial peptides playing anti-inflammatory roles during acute intestinal inflammation^28^ were upregulated upon FMT administration (Fig. 1g). Similarly, a tendency toward upregulation of Muc1 and Muc4, two mucins exerting anti-inflammatory functions in response to pathogens^29^, and a downregulation of Muc3 were observed by FMT treatment in acute DSS-colitis (Fig. 1h).

Thus, our results demonstrate that FMT therapeutic administration during experimental acute colitis ameliorates intestinal inflammation.

### FMT induces variations in the microbial communities

**Fig. 2:**
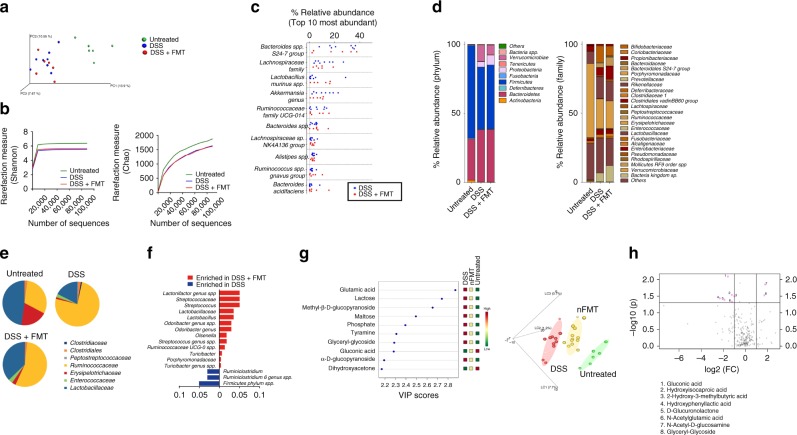
Gut microbiota analysis upon FMT treatment in colitic mice. **a** Microbiome clustering based on unweighted Principal Coordinate Analysis (PCA) UniFrac metrics of fecal gut microbiota derived from DSS treated (blue dots), DSS + FMT-treated (orange dots) and untreated (red dots) mice. **b** Relative abundance of the top 10 most abundant OTUs in DSS (blue) and DSS + FMT (red) treated mice. **c** Rarefaction curves showing microbial richness based on the Chao1 index (bottom panel) and microbial richness and evenness on the Shannon index (upper panel). Green lines, untreated, blue lines, DSS treated; red lines, DSS + FMT-treated samples. **d** Bar plots of the taxonomic composition showing relative abundances > 1% of bacterial phyla (d, left panel) and families (**d**, right panel). **e** Pie charts showing the relative abundance of the less abundant families belonging to the Firmicutes phylum. **f** Comparison of the relative abundances of different taxa between DSS (blue) and DSS + FMT (red) treated mice. Statistical significant difference was assessed through One-way ANOVA with LSD post-hoc test *p < 0.05, **p < 0.01. **g**, **h** Partial Least square-discrimination analysis (PLSD-DA, g) and volcano plot (h) on metabolomics data (g, right panel) and heat map of metabolites that have contributed most to class separation (g, left panel) of faecal samples of untreated, DSS and DSS + FMT-treated mice. Statistical significant difference was assessed through Mann–Whitney. In (**a**–**f**) UT *n* = 6, DSS *n* = 8, DSS + FMT *n* = 6. In (**g**, **h**) UT *n* = 6, DSS *n* = 11, DSS + FMT *n* = 13

Next, variations in the microbial communities in colitic mice treated or not with FMT were evaluated. Fecal samples of DSS and DSS + FMT mice were collected at day 11 post colitis induction and subjected to microbiome profiling using 16S rRNA gene sequencing on the Illumina MiSeq platform. An unweighted UniFrac-based comparison of the microbiota isolated from untreated, DSS and DSS + FMT-treated recipient mice was performed (Fig. 2a and Supplementary figure 4a). Principle component analysis (PCA) differentiated untreated mice from the experimental groups (DSS and DSS + FMT). The intestinal microbiota of recipient mice receiving or not a FMT did not macroscopically differ at sacrifice, possibly due to similar relative abundances of the top 10 most abundant species among DSS and DSS + FMT-derived samples (Fig. 2b). Similarly, the microbiota isolated from DSS-treated mice did not show a lower α-diversity when compared to DSS + FMT-derived microbiota, as reflected by the Chao1 and Shannon indexes (Fig. 2c).

Nonetheless, a detailed phylogenetic analysis of the taxonomic composition of the microbiome of colitic mice treated or not with FMT showed that the reduced inflammatory conditions observed upon FMT administration were associated, with variations in the abundance of specific taxa, including *Firmicutes* and *Verrucomicrobiae*^30,31^. (Fig. 2d–f).

Significant changes towards restoration of normobiosis were detected among the less abundant families belonging to the *Firmicutes* phylum in the DSS + FMT–derived microbiota. For instance, *Clostridiaceae* and *Clostridiales*, which were expanded in colitic mice, were reduced upon FMT and returned to levels comparable to those observed in untreated mice (Fig. 2e).

FMT-derived samples showed significant increases of known commensals used in probiotics preparations^32^, including *Lactobacillaceae* and *Streptococcus sp*., and of the SCFA-producing taxa *Erysipelotrichaceae*, *Ruminococcaceae, Odoribacter* and *Olsenella* (Fig. 2f and Supp Fig. 4B), which are reported to be reduced in IBD patients^4,33^.

Of note, metabolomic analysis showed an increased faecal content of complex sugars including lactose and maltose in DSS-treated mice, a possible consequence of impaired digestion or defective intestinal absorption^34^, whose levels were normalized by FMT-treatment. Similarly, glutamic acid, a metabolite altered in IBD patients^35^, decreased upon FMT while gluconic acid and Dihydroxiacetone, involved in natural detoxification activities^36^, increased upon FMT (Fig. 2g, h).

Altogether, these findings suggest that the beneficial effects of FMT during intestinal inflammation are associated with a reshuffling of the microbiota communities towards restoration of functional normobiosis.

### FMT effects rely on the presence of normobiotic ecologies

Once documented the beneficial effects of FMT, the impact of the donor microbiota composition on the resolution of intestinal inflammation was evaluated.

To do so, mucus and faecal samples were obtained from normobiotic or dysbiotic mice, *i.e* from healthy mice left untreated or treated for 7 days with DSS, and FMT was performed in colitic mice as previously described in Fig. 1a.

**Fig. 3:**
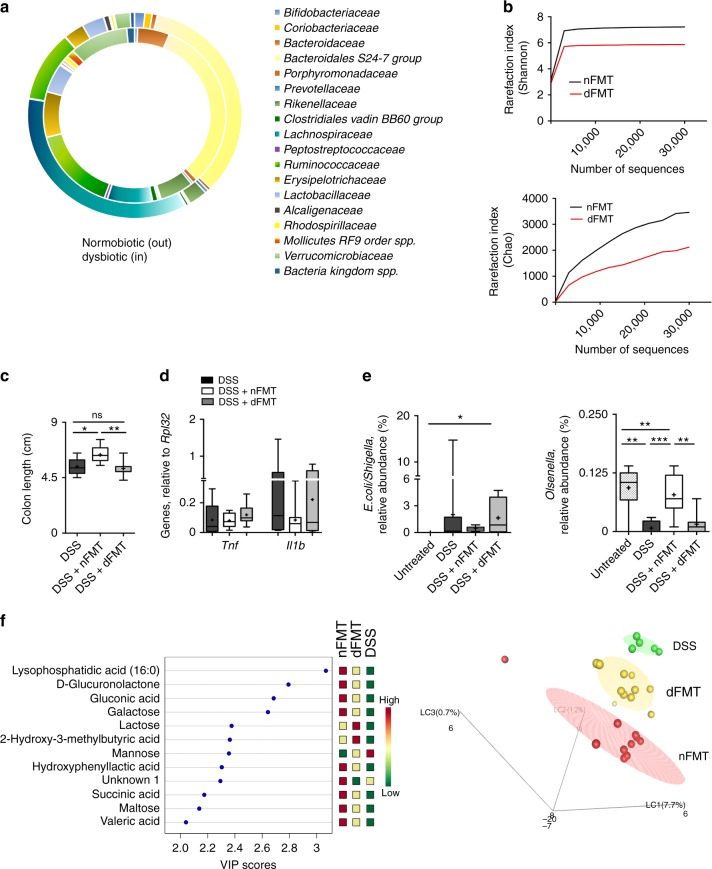
FMT effects upon transfer of normobiotic or dysbiotic ecologies. **a** Comparison of relative abundancies of different taxa between normobiotic (outer chart) and dysbiotic (inner chart) faecal microbiota. **b** Rarefaction curves showing microbial richness based on the Chao1 index (right panel) and microbial richness and evenness on the Shannon index (left panel) Black curves, normal FMT samples, red curves, dysbiotic FMT samples. **c** Colon length of DSS (black boxes), DSS + nFMT (white boxes) or DSS + dFMT (gray boxes) treated mice. **d** Colonic expression of *Tnf* and *Il1b* in DSS (black boxes), DSS + nFMT (white boxes) or DSS + dFMT (gray boxes) treated mice. **e** Relative abundance of E.Coli/Shigella (left panel) and Olsenella (right panel) in untreated (dotted white boxes), DSS treated (black boxes), DSS + nFMT (white boxes) and DSS + dFMT (gray boxes) mice. **f** Partial Least square-discrimination analysis (PLSD-DA) on metabolomics data (right panel) and heat map of metabolites that have contributed most to class separation (left panel) of faecal samples of DSS, DSS + nFMT and DSS + dFMT treated mice. Statistical significance was calculated using a Mann–Whitney test for comparison within two groups or Kruskal–Wallis test with Dunn’s multiple comparison correction within more than two groups. *P < 0.05, **P < 0.01, ***P < 0.001 were regarded as statistically significant. Outliers were detected with Grubb’s test. Non parametric distributions were represented as median+/− interquartile range. In Box and whiskers plots, centre line represents median; cross, represents mean. In (**a**, **b**) nFMT *n* = 7, dFMT *n* = 7. In (**c**, **d**) DSS *n* = 7, DSS + nFMT *n* = 13, DSS + dFMT *n* = 7. In (**e**) UT *n* = 6, DSS *n* = 8, DSS + nFMT *n* = 7, DSS + dFMT *n* = 7. In (**f**) DSS *n* = 5, DSS + nFMT *n* = 10, DSS + dFMT *n* = 11

Relevant differences between the two types of donors used for FMT experiments were confirmed by metagenomic analyses (Fig. 3a, b and Supplementary fig 5A). As previously shown^31^, the microbiota of dysbiotic mice was characterized by a contraction of *Bacteroidales S24-7*, *Lachnospiraceae* and *Bifidobacteriaceae* and an expansion of *Enterobacteriaceae* and *Bacteriaceae* as compared to that of normobiotic mice (Fig. 3a).

As a consequence, an amelioration of the intestinal inflammation was reported in mice receiving a normobiotic FMT but not in those receiving a dysbiotic FMT, as demonstrated by an increase in colon length (Fig. 3c) and a decrease, albeit not significant, in the expression levels of colonic pro-inflammatory *Il1b* and *Tnf* (Fig. 3d).

Consistently, the microbial composition of recipient mice that were transplanted with a dysbiotic FMT was also more similar to that of DSS-treated mice, enriched in pathobionts such as *E.Coli/Shigella* (Fig. 3e), while the microbiota of mice receiving a normobiotic FMT were more similar to that of untreated mice, enriched in protective SCFA-producing bacteria (Fig. 3e). Similarly, metabolomic analysis of the faeces of recipient mice treated with normobiotic FMT revealed the presence of metabolites associated to scavenging of free radicals (d-glucunolactone) and metals (gluconic acid)^36^, to the control of ROS production and neutrophils activity (hydroxiphenillactic acid) and chemotaxis (LPA)^37^, and to SCFA production (Valeric acid)^38^ (Fig. 3f).

**Fig. 4:**
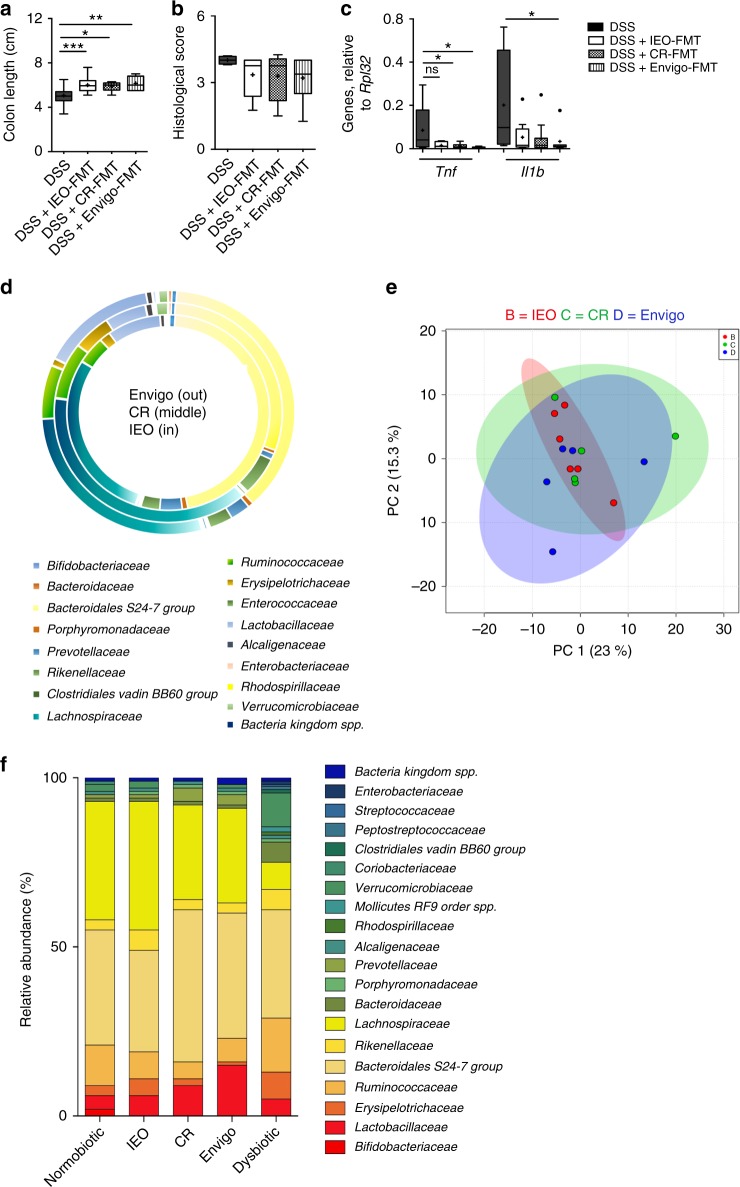
Similar ecologies from different FM donors elicit same beneficial effects. **a**, **b** Colon length (**a**) and cumulative histological score (**b**) of DSS (black boxes), DSS + IEO-FMT (white boxes), DSS + CR-FMT (dotted boxes) and DSS + Envigo-FMT (striped boxes) treated mice. **c** Colonic expression of *Tnf* and *Il1b* in DSS (black boxes), DSS + IEO-FMT (white boxes), DSS + CR-FMT (dotted boxes) and DSS + Envigo-FMT (striped boxes) treated mice **d** Comparison of relative abundancies of different taxa between Envigo- (outer chart), Charles River- (CR) (middle chart) or IEO-derived faecal microbiota. **e** Partial Least square-discrimination analysis (PLSD-DA) on metabolomics data between Envigo- (blue), Charles River- (CR, green) or IEO (red)-derived faecal microbiota. **f** Comparison of relative abundancies of different taxa between normobiotic-, IEO-, CR- Envigo- and dysbiotic-derived fecal microbiota. Statistical significance was calculated using a Mann–Whitney test for comparison within two groups or Kruskal–Wallis test with Dunn’s multiple comparison correction within more than two groups. *P < 0.05, **P < 0.01, ***P < 0.001 were regarded as statistically significant. Outliers were detected with Grubb’s test. Non parametric distributions were represented as median + /- interquartile range. In Box and whiskers plots, centre line represents median; cross, represents mean; dots represent outliers. In (**a**–**c**) DSS *n* = 7, DSS + IEO-FMT *n* = 10, DSS + CR-FMT *n* = 10, DSS + Envigo-FMT *n* = 10. In (**d**, **f**) IEO-FMT *n* = 9, CR-FMT *n* = 10, Envigo-FMT *n* = 9, nFMT = 7, dFMT = 7. In (**e**) DSS *n* = 5, DSS + IEO-FMT *n* = 6, DSS + CR-FMT *n* = 5, DSS + Envigo-FMT *n* = 5

Next, we evaluated if normobiotic donors of different origins might be equally capable to control intestinal inflammation when transplanted into colitic mice. Mucus and faecal samples were isolated form age- and sex-matched C57Bl/6 mice obtained from different sources, *i.e*. from two commercial animal vendors (Charles River srl and Envigo srl) and from in-house bred C57Bl/6 wild-type colony (IEO animal facility). Interestingly, the FMT performed with the microbiota isolated from the different normobiotic donors was equally capable to control intestinal inflammation, as shown by similar colon length (Fig. 4a), histological score (4b) and expression of colonic pro-inflammatory genes (Fig. 4c)

As previously observed^11,39,40^, donor mice sharing the same genetic background, sex and age, but raised in different animal facilities harbored a microbiota genetically similar, not identical, (Fig. 4d, and Supplementary fig 5b) but capable to perform overlapping metabolic activities (Fig. 4e). In accordance to very recent data^41^, 90 to 93% of the total taxa relative abundance of normobiotic mice, regardless of its origin, was composed by a similar core microbial ecology of *Bacteroidales S24-7* (30-45% among groups), *Lachnospiraceae* (28-38%), *Lactobacillaceae* (6-15%), *Ruminococcaceae* (5-8%), *Rikenellaceae* (3−6%), *Bifidobacteriaceae* (1–2%) and *Erysipelotrichaeceae* (1–5%), that in dysbiotic mice accounted only for a total 75% (Fig. 4f). These taxa have been functionally associated to homeostatic metabolic activities, including SCFA production leading to Treg cells differentiation and IL-10-production^42-44^. On the contrary, the taxa expanded in dysbiotic mice accounting for a 25% of the total microbial ecology, which were either completely absent or present in extremely low abundancies in normobiotic mice, were mostly *Enterobacteriaceae*, *Bactaeriaceae*, *Rhodospirillaceae*, *Streptococcaceae*, which have been shown to be increased both in colitic mice and IBD patients^4,31^(Fig. 4f)

Taken together, these data indicate that beneficial effects of FMT are strictly dependent on the graft composition, and that only a healthy microbiota can ameliorate the intestinal inflammation.

### FMT influences colonic immune cells relative abundance

**Fig. 5:**
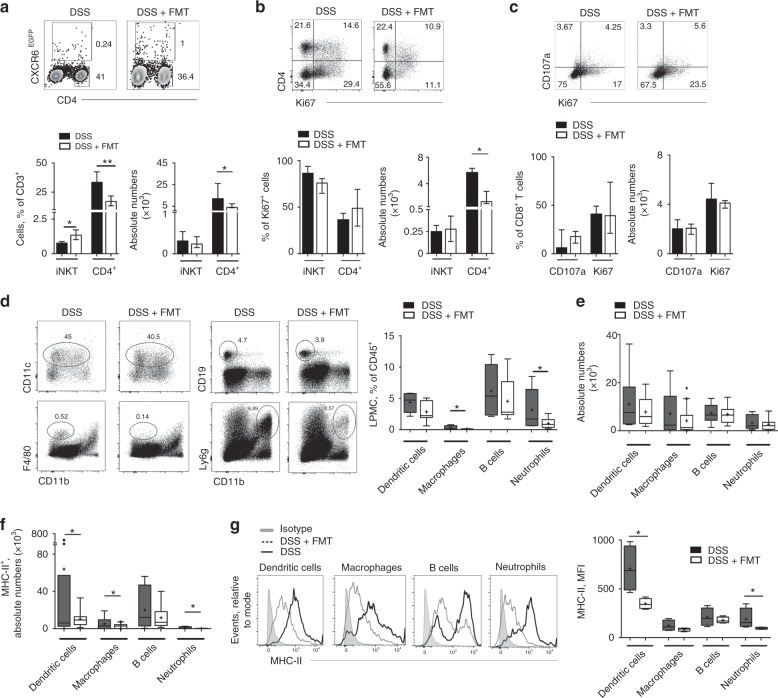
Therapeutic FMT modulates immune cell frequencies and phenotypes. **a** Representative dot plots (upper panels), frequencies and absolute numbers (lower panels) of colonic CD4^+^ T cells and iNKT cells in DSS-treated (black bars) and FMT-treated (white bars) mice 11 days after starting DSS administration. **b** Representative dot plots, (upper panels), frequencies and absolute numbers (lower panels) of Ki67-expressing colonic CD4^+^ T cells and iNKT cells in DSS-treated (black bars) and FMT-treated (white bars) mice. **c** Representative dot plots, (upper panels), frequencies and absolute numbers (lower panels) of Ki67 and CD107a- expressing colonic CD8^+^ T cells in DSS-treated (black bars) and FMT-treated (white bars) mice. **d** Representative dot plots and frequency of dendritic cells (CD45.2^+^CD3^-^CD11c^+^), macrophages (CD45.2^+^CD3^-^F4/80^+^CD11b^−^), B cells (CD45.2^+^CD3^−^CD19^+^), neutrophils (CD45.2^+^CD3^−^Ly6g^+^CD11b^+^) in DSS-treated (left panels) and FMT-treated (right panels) mice 11 days after starting DSS administration. **e** Absolute numbers of colonic dendritic cells, macrophages, B cells, neutrophils in DSS-treated (black boxes) and DSS + FMT-treated (white boxes). **f** Absolute numbers of MHC-II expressing colonic dendritic cells, macrophages, B cells and neutrophils in DSS (white boxes) and DSS + FMT-treated (black boxes) mice. **g** Representative histograms of MHC-II expression and cumulative Mean fluorescence intensity (MFI) on colonic dendritic cells, macrophages, B cells, neutrophils in DSS (black boxes) and DSS + FMT (white boxes) treated mice. Statistical significance was calculated using a Mann–Whitney test for comparison within two groups or Kruskal–Wallis test with Dunn’s multiple comparison correction within more than two groups. *P < 0.05, **P < 0.01, ***P < 0.001 were regarded as statistically significant. Outliers were detected with Grubb’s test. Non parametric distributions were represented as median + /- interquartile range. In Box and whiskers plots, centre line represents median; cross, represents mean; dots represent outliers. In (**a**–**c**) DSS *n* = 12, DSS + FMT *n* = 12. In (**d**–**g**) DSS *n* = 16, DSS + FMT *n* = 16

Given the strict interdependence between intestinal immune system and the host microbiota^45^, we next aimed to explore immune pathways selectively modulated by FMT, We thus asked if therapeutic FMT administration in colitic mice induced variations in the frequencies and in the functional activities of the immune cell colonic infiltrate. The presence of specific bacterial strains in the gut has been linked to the differentiation and expansion of conventional^11^ and unconventional^25^ CD4^+^ T cells. An increase of iNKT cell frequency, but not absolute numbers, (Fig. 5a) was observed in DSS-treated mice after FMT, while CD4^+^ T cells were decreased both in frequency and numbers (Fig. 5a), in line with their reduced proliferative capacity (Fig. 5b). Given that iNKT cells isolated from FMT-treated and untreated mice exhibited similar Ki67 expression (Fig. 5b), the increase of iNKT cell percentages on total CD3^+^ lymphocytes was likely a consequence of the overall reduction of CD4^+^ T cells rather than of their expansion (Fig. 5b). On the contrary, colonic CD8^+^ T cells frequency (Supplementary Fig. 6a) and phenotype (Fig. 5c) were not affected by FMT-treatment.

Interestingly, we found that FMT treatment was able to reduce the inflammation-driven expansion of the innate lymphocytes ILC2 and ILC3^46,47^ in the lamina propria of acute DSS-treated mice (Supplementary Fig. 6b, c)

Antigen presenting cells (APC) and immune cells of myeloid origin infiltrating the inflamed lamina propria have been shown to contribute to sustain and propagate intestinal inflammation^2^. Following therapeutic FMT, a reduction in F4/80^+^ macrophages and CD11b^+^Ly6G^+^ neutrophils frequency (Fig. 5d) and partially in absolute numbers (Fig. 5e) was observed. In contrast, CD19^+^B cells and CD11c^+^dendritic cells were mostly unaffected (Fig. 5d, e).

Further, several evidences suggest that mucosal T-helper cells activation during intestinal inflammation depends on antigenic stimulation^2^. After FMT, both the number of colonic MHC-II-expressing professional APC, including dendritic cells and macrophages, (Fig. 5f) and MHC-II expression levels on APC (Fig. 5g) were strongly reduced, suggesting that FMT might directly act on the antigen presenting capacity of professional APC.

To note, similar findings were observed in the TNBS-induced experimental colitis model (Supplementary Figure 7)

Taken together, these data indicate that therapeutic FMT exerts a specific effect on infiltrating immune cells population frequency and phenotype.

### CD4^+^ T cells cytokine skewing requires antigen presentation

We next evaluated if FMT administration might influence specific immune cells functional activities that are directly correlated to bacterial antigens presentation.

**Fig. 6:**
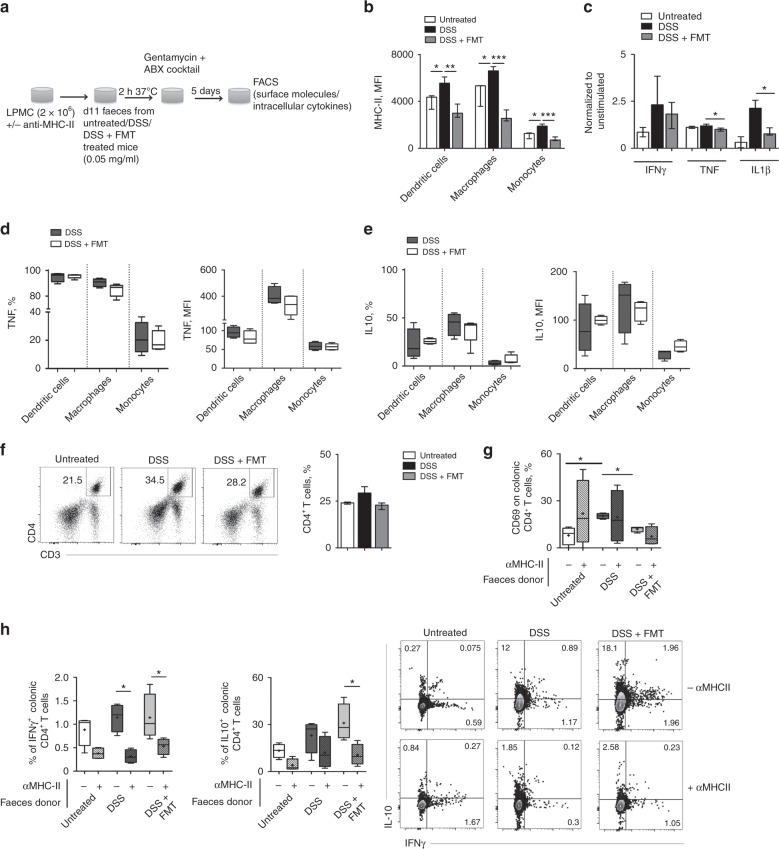
Bacterial antigens presentation is required for CD4^*+*^T cells cytokine skewing. **a** Schematic representation of the experiment. **b** Mean fluorescence intensity (MFI) of MHC-II on dendritic cells, macrophages, monocytes, neutrophils exposed in vitro to faeces isolated from untreated (white bars), DSS (black bars) and DSS + FMT (grey bars) treated mice. **c** IFNg, TNF and IL1b cytokines levels in supernatants of LPMC in vitro exposed to faeces isolated from untreated (white bars), DSS (black bars) and DSS + FMT (grey bars) treated mice. Cytokines levels were normalised to unstimulated spontaneous secretion by LPMC. **d**, **e** Frequencies (left graphs) and MFI (right graphs) of TNF- (**d**) and IL-10- (**e**) secreting dendritic cells, macrophages and monocytes exposed in vitro to faeces isolated from DSS (dark gray bars) and DSS + FMT (white bars) treated mice (**f**) Representative dot plots and frequencies of CD4^+^ T cells exposed in vitro to faeces isolated from untreated (white bars), DSS (black bars) and DSS + FMT (grey bars) treated mice. **g** CD69 expression on intestinal CD4^+^ T cells exposed in vitro to faeces isolated from untreated (white boxes), DSS (black boxes) and DSS + FMT (grey boxes) treated mice in the presence (striped bars) or absence (filled bars) of blocking αMHC-II antibodies. **h** Frequencies and representative dot plots of IFNγ and IL-10 secreting intestinal CD4^+^ T cells exposed in vitro to faeces isolated from untreated (white bars), DSS (black bars) and DSS + FMT (grey bars) treated mice in the presence (striped bars) or absence (filled bars) of blocking αMHC-II antibodies. Statistical significance was calculated using a Mann–Whitney test for comparison within two groups or Kruskal–Wallis test with Dunn’s multiple comparison correction within more than two groups. *P < 0.05, **P < 0.01, ***P < 0.001 were regarded as statistically significant. Outliers were detected with Grubb’s test. Non parametric distributions were represented as median + /− interquartile range. In Box and whiskers plots, centre line represents median; cross, represents mean; dots represent outliers. In all panels UT *n* = 5, DSS *n* = 7, DSS + FMT *n* = 9

The faeces of untreated, DSS-treated and FMT-treated mice were collected and used to stimulate in-vitro intestinal lamina propria mononuclear cells (LPMC) freshly isolated from healthy mice (Fig. 6a).

As observed in vivo, MHC-II levels on dendritic cells, macrophages and monocytes were down-regulated upon in vitro exposure to normal (untreated) or FMT-derived microbiota (Fig. 6b). Similarly, the expression of CD86, a co-stimulatory molecule critically involved in T cell stimulation, was reduced on APC exposed to untreated or FMT-derived microbiota as compared to those exposed to the faeces of DSS-treated mice (Supplementary Fig. 8a).

Cytokines produced by mucosal antigen presenting cells play a pivotal role in the initiation and propagation of intestinal inflammation, as in the maintenance of homeostasis^48^. To evaluate the cytokine milieu generated upon exposure to FMT-derived microbiota, we analysed the supernatants of stimulated intestinal LPMC (Fig. 6c). Interestingly, LPMC exposed to FMT-derived microbiota showed reduced levels of pro-inflammatory cytokines, such as TNF, IL1β and IFNγ (Fig. 6c), which were instead increased in the supernatants of LPMC exposed to DSS-derived microbiota.

FMT-derived microbiota was also capable to differently skew the cytokine profile of antigen presenting cells (Fig. 6d, e). Indeed, the frequency of pro-inflammatory TNF-producing intestinal dendritic cells and macrophages was strongly reduced upon exposure to FMT-derived microbiota (Fig. 6d). Conversely, FMT-derived microbiota increased the frequency of IL-10-producing intestinal dendritic cells and monocytes (Fig. 6e).

Correspondingly, CD4^+^ T cell expansion occurred only after stimulation with DSS-derived, but not with normal or FMT-derived microbiota (Fig. 6f). To note, FMT-derived microbiota slightly increased the frequency of Foxp3 + Treg cells (Supplementary Fig. 8b).

DSS-derived microbiota also induced a strong upregulation of CD69 on intestinal CD4^+^ T cells, while untreated or FMT-derived microbiota failed to do so (Fig. 6g). Likewise, only FMT-derived microbiota significantly increased IL-10 secretion by CD4 + T cells, in an MHC-II dependent fashion (Fig. 6h). Importantly, also IFNγ secretion was dependent on antigen presentation (Fig. 6h).

Taken together these data confirm a crucial role for bacterial antigen presentation in the tolerogenic skewing of innate and adaptive colonic immune populations upon FMT treatment.

### IL-10 critically contributes to FMT beneficial effects

In vitro experiments indicated that IL-10 production by intestinal immune cells might be critically involved in the tolerogenic mechanisms triggered by therapeutic FMT during experimental colitis. We thus evaluated if these mechanisms occurred in vivo upon FMT, and if IL-10 production by immune cells might be responsible for the observed therapeutic effects of FMT.

**Fig. 7:**
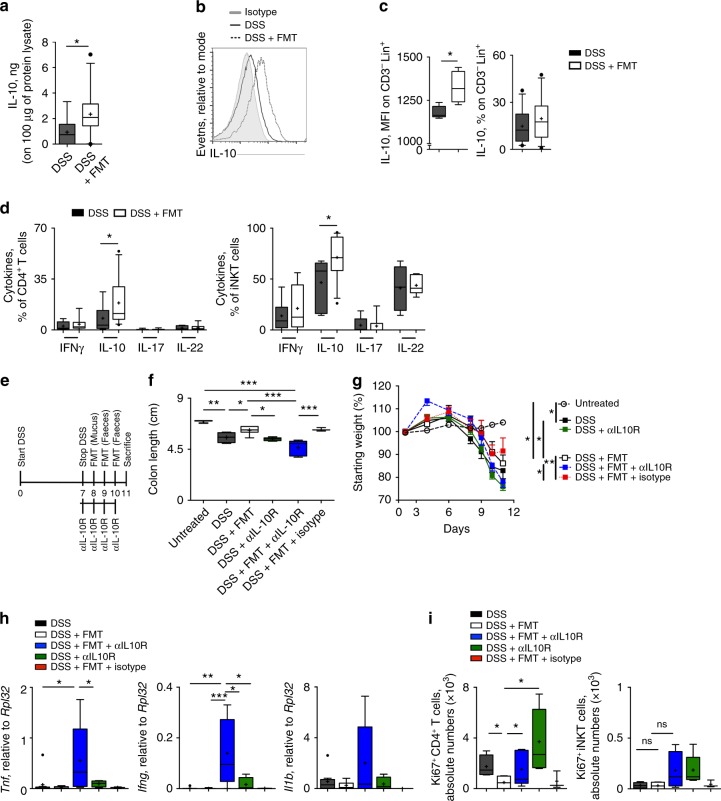
IL-10 contributes to FMT beneficial therapeutic effects. **a** IL-10 colonic expression in DSS-(black boxes) and DSS + FMT-(white boxes) treated mice. **b**, **c** Representative histograms of IL-10 expression (**b**) and cumulative Mean fluorescence intensity (MFI) (**c**, left panel) and frequency (**c**, right panel) of IL-10-producing colonic professional APC (CD45^+^CD3^−^MHC-II^+^) in DSS (black boxes) and DSS + FMT (white boxes) treated mice. **d** Summary of cytokines secreted by colonic CD4^+^ T cells and iNKT cells in DSS-treated (black boxes) and FMT-treated (white boxes) mice 11 days after starting DSS administration. **e** Schematic representation of the experiment. **f**, **g** Colon length (**f**) and weight loss (**g**) of untreated mice (white dotted boxes/open circles), or receiving DSS (black boxes/black symbols), or DSS + FMT (white boxes/white square symbols) or DSS + αIL-10R (green boxes/green symbols), or DSS + FMT + αIL-10R (blue boxes and blue symbols) or mice receiving DSS + FMT + αIL-10R isotype antibody (red boxes/red symbols). **h** Colonic expression of *Tnf, Ifnγ and IL1β* in DSS (black boxes), DSS + FMT (white boxes), DSS + αIL-10R (blue boxes), DSS + FMT + αIL-10R- (green boxes) and DSS + FMT + αIL-10R-isotype-antibody-treated mice (red boxes). **i** Absolute numbers of KI67 expressing colonic CD4^+^ T cells (left panels) and iNKT cells (right panels) in DSS (black boxes), DSS + FMT (white boxes), DSS + αIL-10R (blue boxes), DSS + FMT + αIL-10R (green boxes) and DSS + FMT + αIL-10R isotype antibody (red boxes). Statistical significance was calculated using a Mann–Whitney test for comparison within two groups or Kruskal–Wallis test with Dunn’s multiple comparison correction within more than two groups. *P < 0.05, **P < 0.01, ***P < 0.001 were regarded as statistically significant. Outliers were detected with Grubb’s test. Non parametric distributions were represented as median + /- interquartile range. In Box and whiskers plots, centre line represents median; cross, represents mean; dots represent outliers. In (**a**) DSS *n* = 15,DSS + FMT *n* = 26. In (**b**–**d**) DSS *n* = 14,DSS + FMT *n* = 17. In (**e**–**i**) DSS, DSS + FMT *n* = 4; DSS + αIL-10R,DSS + FMT + αIL-10R *n* = 5, DSS + FMT + isotype *n* = 3

Higher amounts of colonic IL-10 (Fig. 7a) as well as increased frequencies of IL-10-producing APC (Fig. 7b, c) and CD4^+^ T and iNKT cells (Fig. 7d) were observed in the colons of FMT-treated mice as compared to DSS-treated mice. Interestingly, the increased IL-10 secretion by T cells observed in FMT-treated mice normalised upon inflammation resolution (Supplementary Fig. 9b). To test the contribution of IL-10 on the anti-inflammatory properties of FMT, DSS-treated mice were administered FMT concomitantly to IL-10 receptor (IL-10R) blockade (Fig. 7e). Inhibition of the tolerogenic functions of IL-10 on IL-10R expressing cells, such as antigen presenting cells (APC), T cells and epithelial cells^49^, hampered FMT protective effects as shown by reduced colon length (Fig. 7f), increased weight loss (Fig. 7g) and higher colonic expression of *Tnf, Ifnγ* and *Il1β* (Fig. 7h). These effects were not observed when IL-10R was blocked in colitic mice without a concomitant FMT administration, suggesting a direct contribution of the microflora in the IL-10-mediated control of inflammation. As expected, IL-10R blockade reverted the inhibition of CD4^+^ T cells proliferation occurring upon FMT (Fig. 7i). IL-10R blockade also affected IL-10 production by T and iNKT cells, possibly through a feedback regulatory loop (Supplementary Fig 9c).

### Protective bacteria selective elimination impairs FMT effect

**Fig. 8:**
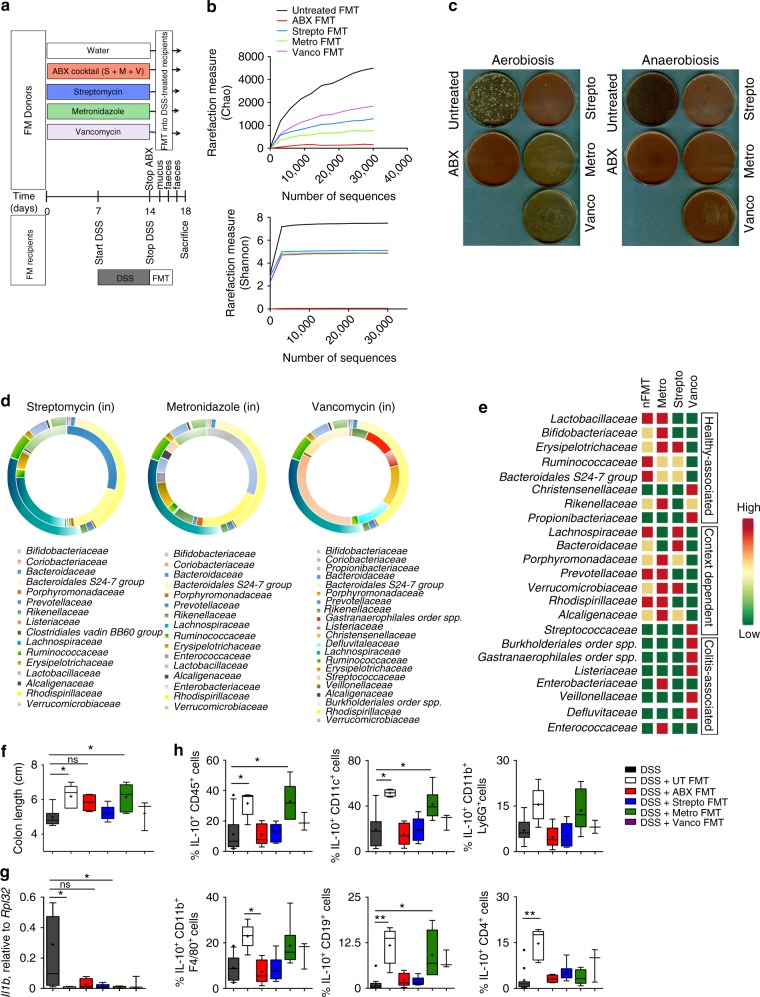
Antibiotic treatment selectively abolishes FMT beneficial effects. **a** Schematic representation of the experiment. **b** Rarefaction curves showing microbial richness (Chao1 index,top panel) and microbial richness and evenness (Shannon index,bottom panel). Black line, untreated-, red line ABXFMT-, blue line Strepto FMT-, Green line Metro FMT-, Violet line Vanco FMT- derived samples. **c** Plating of faecal material derived from untreated, treated with the antibiotic cocktail (ABX) or with streptomycin (Strepto), Metronidazole (Metro) or Vancomycin (Vanco) in aerobiosis (left) and anaerobiosis (right). **d** Comparison of relative abundancies of different taxa between faecal microbiota obtained from normobiotic (outer chart) and Streptomycin-treated mice (inner chart, left panel), or Metronidazole-treated (inner chart, middle panel) or Vancomycin-treated mice(inner chart, right panel). **e** Heat map comparing the expression levels of the different taxa between faecal microbiota obtained from normobiotic, Streptomycin-, Metronidazole- or Vancomycin-treated mice. **f**, **g** Colon length (**f**) and *il1b* colonic expression (**g**) in DSS-treated (black boxes), DSS + Untreated FMT (white boxes), DSS + ABX FMT (red boxes), DSS + Streptomycin FMT (blue boxes), DSS + Metronidazole FMT (green boxes) and Vancomycin (Violet boxes)-treated mice. **h** Frequencies IL-10 secreting colonic total CD45 + immune cell populations or gated dendritic cells (CD11c + ), neutrophils (Cd11b + Ly6g + ), Macrophages (Cd11b + F4/80 + ), B cells (CD19 + ) and CD4^+^ T cells isolated from DSS-treated (black boxes), DSS + untreated FMT (white boxes), DSS + ABX FMT (red boxes), DSS + Streptomycin FMT (blue boxes), DSS + Metronidazole FMT (green boxes) and DSS + Vancomycin FMT (Violet boxes)-treated mice. Statistical significance was calculated using a Mann–Whitney test for comparison within two groups or Kruskal–Wallis test with Dunn’s multiple comparison correction within more than two groups. *P < 0.05, **P < 0.01, ***P < 0.001 were regarded as statistically significant. Outliers were detected with Grubb’s test. Non parametric distributions were represented as median+/− interquartile range. In Box and whiskers plots, centre line represents median; cross, represents mean; dots represent outliers. In (**b**–**e**) UT *n* = 4; DSS, DSS + Abx FMT *n* = 5; DSS + Strepto FMT, DSS + Metro FMT *n* = 6; DSS + FMT Vanco *n* = 3. In (**f**–**h**) UT *n* = 4; DSS *n* = 5; DSS + Abx FMT, DSS + Strepto FMT, DSS + Metro FMT, DSS + FMT Vanco *n* = 6

To further correlate the gut microbiota composition to the induction of protective immune-mediated functions during intestinal inflammation, therapeutic FMT was performed with mucus and faeces isolated from mice previously pre-treated for two weeks with different antibiotics targeting either Gram-positive organisms (vancomycin), Gram- negative bacteria (streptomycin), strict anaerobes (metronidazole) or having a broad spectrum bacterial depletion capability (ABX) (Fig. 8a)^50^.

The taxonomic composition of the different donor microbiota was analysed by 16 S rRNA gene sequencing before administration to colitic mice. As expected, the α-diversity in antibiotic-treated samples was lower as compared to those derived from normobiotic samples (Fig. 8b), evidence confirmed also by plating of the faecal material in aerobic and anaerobic conditions (Fig. 8c).

The detailed phylogenetic analysis of the taxonomic composition highlighted a relevant dysbiosis in the antibiotic- treated donor samples as compared to the untreated ones (Fig. 8d, e). In particular, metronidazole treatment favored the selective persistence of *Lactobacillaceae*, *Bifidobacteriaceae* and *Erysipelotrichaceae*, as well as of *Ruminococcacceae* and *Bacteroidales S24-7*, families belonging to the protective normobiotic microbial ecologies^31^ previously described in Fig. 4. On the contrary, pathobionts such as *Christensenellaceae*, *Burkholderiales*, *Listeriaceae* and *Gastranaerophilales* significantly emerged in Vancomycin-treated samples at the expenses of the abovementioned protective families. Streptomycin-treatment, instead, favoured a limited presence of *Erysipelotrichaceae* and *Ruminococcaceae* while not expanding pathobionts, but rather allowing the survival of families whose function could be protective or detrimental according to the context^1,51^. As a proof of concept, the microbial population of broad spectrum antibiotic-treated mice was completely depleted, as confirmed by the plating of their faecal material (Fig. 8c).

Of note, FMT performed with metronidazole-treated microbiota retained a full capability to control intestinal inflammation (Fig. 8f, g and Supplementary Fig 10). Importantly, this was associated with a selective increased production of IL-10 by colonic APC (Fig. 8h), as to confirm the previously suggested IL-10 promoting activity of *Lactobacillaceae* and B*ifidobacteriaceae*^52^.

**Fig. 9:**
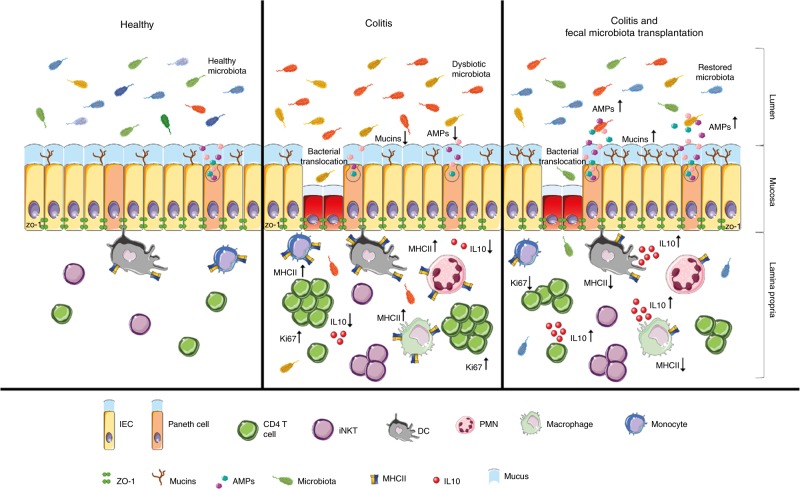
Graphical abstract of events occurring in the intestinal mucosa in healthy conditions, during intestinal inflammation caused by DSS administration and during faecal transplantation in colitic mice. Adapted from SERVIER MEDICAL ART (CC BY 3.0 Licence)

In conclusion, our data show that the gut microbiome modifications after FMT exert a profound impact on the mucosal immune system. The composition of the microbial ecology transferred by FMT is pivotal to its beneficial anti-inflammatory effects by supporting changes in immune cell frequencies, the reduction of colonic *ifnγ* and *il1β*, the increase in antimicrobial peptides and mucins, and the decrease of bacterial antigen presentation by APC. Most importantly, normobiotic FMT induces the skewing of innate and adaptive immune cells toward a tolerogenic IL-10 secreting cytokine profile that, altogether, concur to restore intestinal homeostasis (Fig. 9).

## Discussion

FMT is becoming the first-line therapy in antibiotic resistant recurrent CDI^14,15^. However, its therapeutic application to other gastrointestinal diseases is at the very beginning and data on its mechanism of action during intestinal inflammation are still scarce. While it is known that restoration of normobiosis correlates with clinical remission in successful trials involving UC patients^16–18^, it is still unclear whether FMT might have an effect on the immune system. To date, data in BALB/c mice suggest that FMT can induce CD4^+^CD25^+^ regulatory T cells and reduce colonic expression of *Il1β* and *Ifnγ*^19^.

In our study, we show for the first time that the manipulation of the gut microbiota by FMT induces variations in both innate and adaptive immune cell frequencies and cytokine profiles and that this correlates with a general amelioration of the inflammatory status in colitic animals. Moreover, analyses of the gut microbiota in our model showed that 3 days of therapeutic FMT are sufficient to introduce modifications in the dysbiotic microbiota, with a relevant change in the relative proportions of *Firmicutes*. These results are in agreement with findings from successful FMT clinical trials on UC patients and from animal models of intestinal inflammation. In particular, data in UC patients showed that clinical remission correlated with stable modifications of the gut microbiota towards functional normobiosis restoration^16–18^ and that this shift in intestinal microbial ecology was sufficient to trigger reduction of inflammatory genes such as *ifnγ, il1β* and *tnf*^53^. Further, in IBD patients and in animal models of intestinal inflammation^4,33,54^ changes in the levels of *Erysipelotrichaceae* and *Lactobacillacee* have been described. Interestingly, we found that these taxa were also similarly altered in DSS-treated mice, and their abundances were restored upon therapeutically successful FMT.

It is now acknowledged that the gut microbiota composition is heavily influenced by age, gender, genotype, diet and environmental factors^55^. In this context, animal models are valuable tools to study complex biological phenomena, such as those occurring during intestinal inflammatory processes, while controlling confounding variables. It also emerged from several recent studies that different research institutions or commercial vendors might harbour variations in the microbiological environment^39,40^, leading to differences in microbiota composition. Here we showed that the microbiota derived from three different sources were equally capable to control intestinal inflammation in our experimental model, and this capability was dependent on the presence of a core microbial ecology composed by *Bacteroidales S24-7*, *Lachnospiraceae*, *Lactobacillaceae*, *Ruminococcaceae*, *Rikenellaceae*, *Bifidobacteriaceae* and *Erysipelotrichaceae*. As recently shown also in metabolic diseases^41^ and in murine models of intestinal inflammation^31^, this core ecology consists of bacteria from different phyla sharing similar metabolic functions (i.e: SCFA production, Ph control, free radicals scavenging) and, when maintained at a certain population level, capable to create environmental conditions sufficient to inhibit the growth of pathogenic species/pathobionts and support optimal host health.

A healthy gut environment, shaped by the presence of a healthy functional microbial ecosystem, is thus fundamental to instruct the immune system towards homeostasis.

Several reports describe the pivotal role of T cells as key players in the initiation and maintenance of intestinal inflammation. For instance, we^56^ and others^57,58^ reported that IBD patients show increased amounts of intestinal Th1, Th17 and Th1/17 cells whose pathogenic role has been associated to the recognition of bacterial antigens^7,59,60^. In addition to conventional CD4^+^ T cells, other T cell subsets, like iNKT cells, can secrete IL17A and IFNγ in the gut and affect bacterial colonization of the intestine^20,61^ but their role in intestinal inflammation is less well defined^62^.

In our study, in vitro stimulation of intestinal lamina propria cells with faecal content from untreated, DSS or FMT-treated mice induced a different cytokine profile in both innate and adaptive immune cells, suggesting that the alterations in the microbiota ecology during colitis and upon FMT are directly linked to changes in the functional status of the mucosal immune system.

Additionally, our data indicate that variations in the intestinal microbial ecology are also capable to skew, both in vitro and in vivo, the cytokine profile of antigen presenting cells from pro-inflammatory to tolerogenic^45^.

In particular, we show that in acute models of intestinal inflammation, IL-10 secretion by APC and T cells upon FMT is temporally linked to the resolution of inflammation, confirming a direct contribution of IL-10-mediated functional activities in intestinal homeostasis^63^ also in the context of FMT. IL-10 is an anti-inflammatory cytokine known to critically maintain intestinal immune homeostasis^64^. IL-10-deficient mice and patients with genetic defects in the IL-10/IL-10R pathway develop intestinal inflammation in the presence of a normal gut microflora. Moreover, the presence of specific commensal bacteria such as *Lactobacilli* and *Bifidobacteria* have been directly associated to the secretion of tolerogenic IL-10^52^ and has been implicated in the maintenance of intestinal homeostasis^65^. Several studies report that a probiotic preparation (#VSL3) composed by a cocktail of eight strains of *Lactobacilli* and *Bifidobacteria* is effective in controlling intestinal inflammation^32^

The resolution of intestinal inflammation has been also linked to the activation and the functional plasticity of CD4^+^ T cells from pro-inflammatory to tolerogenic/IL-10-producing subtypes^63,66^. Upon therapeutic FMT, both CD4 + and iNKT cells produce IL-10. Importantly, since IL-10 also negatively affects the proliferative capacity of cells the overall increased availability of colonic IL-10 might be directly responsible for the reduced proliferative capacity of T cells together with the decreased antigen presentation by APC, as shown by their reduced levels of MHC-II.

In this manuscript we show that colonic APC, such as neutrophils and macrophages, both recruited in the inflamed gut^67,68^ are also strongly reduced in frequency and absolute number upon therapeutic FMT. This is in line with findings showing that macrophages contribute to IBD pathogenesis and are direct target of anti-TNF therapy, which induces their apoptosis^69^ and/or a regulatory M2 phenotype^67^.

Finally, we also show that the levels of MHCII and the frequency of MHCII^+^ professional APC in the colonic lamina propria are strongly down-regulated upon FMT. Importantly, colonic HLA-DR expression levels discriminate between healthy, quiescent and active IBD patients^70^, confirming a prominent role of MHCII-dependent antigen presentation in IBD immunopathology. Since IFNγ up-regulates MHCII molecules, the observed reduction of colonic IFNγ after FMT could explain the decrease of MHCII surface levels and it might be instrumental for reducing the presentation of bacterial antigens to CD4^+^T cells.

In conclusion, we demonstrate that modulation of intestinal microbiota by FMT during experimental colitis exerts multiple effects in both adaptive and innate mucosal immune responses. The restoration of normobiosis, possibly also through cooperative interactions among commensal species, could be the first hint to simultaneously trigger several immune pathways leading to tolerogenic functions of innate and adaptive immune cells that altogether contribute to the resolution of the inflammatory processes. Further studies will highlight if defined microbial species or community-based effects contribute to tolerogenic mechanisms in the gut.

These findings on overall represent an important contribution toward the elucidation of the complex interplay between the immune system and the gut microbial ecosystem and are instrumental for better understanding the immune events occurring during therapeutic FMT in humans.

## Methods

### Mice

C57BL/6 mice (Charles River, IT) and CXCR6 EGFP/ + mice (B6.129P2-*Cxcr6tm1Litt*/J; IMSR_JAX: 005693) background C57BL/6, purchased as GFP/GFP from JAX, USA, and bred to heterozigosity with C57BL/6 mice) of 8–10 weeks of age were housed at the IEO animal facility in SPF conditions. Experimental groups of mice receiving the different FMT treatments were kept in separated cages. Littermates of same sex and age were randomly assigned to the different experimental groups.

Animal procedures were approved by the Italian Ministry of Health (Auth. 127/15, 27/13, 913/16, 415/17) and by the OPBA of the European Institute of Oncology, IEO, Italy.

### Experimental colitis models

For the induction of DSS-induced acute colitis, mice were given 2% (w/v) dextran sodium sulphate (DSS, MW 40 kD; TdB Consultancy) in their drinking water for 7 days followed by 2 days of recovery. The weight curve was determined by weighing mice daily. At sacrifice, colons were collected, their length was measured and divided in portions to be fixed in 10% formalin for histological analyses, snap-frozen for RNA extraction and for lamina propria mononuclear cells (LPMC) immunophenotyping.

### Faecal microbiota transplantation (FMT)

FMT was performed through oral gavage of mucus (first day) and faeces (second and third days) preparations from donor mice. This protocol facilitates the engraftment of the mucus-associated bacteria. Donor mice were untreated (normobiotic) donors, DSS-treated (dysbiotic) or treated with metronidazole (1 g/L), vancomycin (1 g/L), streptomycin (2 g/L) or a cocktail of the three antibiotics combined in order to deplete distinct taxa of bacteria. Mucus was scraped from colons, diluted in PBS and administered to recipients at 1:1 ratio. Faeces were collected, diluted in PBS (50 mg/ml) and administered to recipients by oral gavage (10 mg/mouse) one day after the end of acute DSS administration.

For in vivo IL-10R blockade, mice were injected intraperitoneally with 250 µg InVivoMAb anti-mouse IL-10R (BioXCell, clone 1B1.3 A), or its Isotype (BioXCell), daily for 4 days starting from one day before FMT treatment.

### Murine cell isolation

For LPMC isolation, Peyer’s Patches were removed, colonic lamina propria lymphocytes (LPL) were isolated via incubation with 5 mM EDTA at 37 °C for 30 min, followed by further digestion with collagenase IV and DNase at 37 °C for 1 h. Cells were then separated with a Percoll gradient.

In some experiments after isolation cells were re-stimulated in vitro for 3 h with PMA/Ionomycin in the presence of Brefeldin A for cytokine secretion.

### Flow cytometry analysis

Cells were stained with combinations of directly conjugated antibodies described in Supplementary Table 3. iNKT cells were identified by CXCR6-^EGFP^ expression or mCD1d:PBS57 Tetramer (NIH Tetramer core facility) staining.

Intracellular staining of cytokines was performed after cells fixtion and permeabilization with Cytofix/Cytoperm (BD) before addition of the antibodies. Samples were analysed by a FACSCanto II flow cytometer (BD), gated to exclude nonviable cells. Data were analysed using FlowJo software (BD). The complete list of the antibodies and dyes used in the study are described in Supplementary Table 3.

### RT-qPCR of tissue mRNA

Total RNA from colonic tissues was isolated using TRIZOL and Quick-RNA MiniPrep (ZymoResearch) according to manufacturer’s specifications and following the *MetaHIT* project guidelines. cDNAs were generated from 1 µg of total RNA with reverse transcription kit (Promega). Gene expression levels were evaluated by qPCR and normalized to *Rpl32* gene expression. The primer sequences are collected in Supplementary Table 2.

### Immunofluorescence

Intestinal samples were fixed overnight in paraformaldehyde, l-Lysine pH 7.4 and NaIO4 (PLP buffer). They were then washed, dehydrated in 20% sucrose for at least 4 h and included in OCT (Sakura). 10μm-thick sections were re-hydrated with 0.1 M Tris HCl pH: 7.4 buffer and blocked with 0.3%triton X-100, 2% FBS 0.1 M Tris-HCl buffer. Slides were incubated with the primary antibody (anti ZO-1 FITC, 1:100) for 2 hrs. Nuclei were counterstained with DAPI (1:30.000; Roche) and mounted with Vectashield (Vectorlabs).

### Histological analysis

Tissue processing was performed with a LEICA PELORIS processor before paraffin embedding^71^. Murine samples were included using an automated system (SAKURA Tissue-Tek). After Hematoxylin and Eosin staining, snapshots of histology were taken using an Aperio CS2 microscope with a scanning resolution of 50,000 pixels per inch (0.5 µm per pixel with 10x objective and 2.5 µm per pixel when scanning at ×4). Scoring of disease activity was performed according to the criteria described in Supplementary Table 1.

### Microbiota identification

Faeces and mucus scraped from the colon were stored at −80 °C until the DNA was extracted with G NOME DNA isolation kit (MP) following the protocol described in^72^ Partial 16 S rRNA gene sequences were amplified using primer pair Probio_Uni and /Probio_Rev, targeting the V3 region of the 16 S rRNA gene sequence^73^. The 16S rRNA gene sequencing was performed using a MiSeq (Illumina) at the DNA sequencing facility of GenProbio srl (www.genprobio.com)^73^.

Following sequencing, the obtained individual sequence reads were filtered by the Illumina software to remove low quality and polyclonal sequences. All Illumina quality-approved, trimmed and filtered data were exported as.fastq files. The.fastq files were processed using a custom script based on the QIIME software suite^74^. Quality control retained sequences with a length between 140 and 400 bp and mean sequence quality score > 20 while sequences with homopolymers > 7 bp and mismatched primers were omitted. To calculate downstream diversity measures (alpha and beta diversity indices, Unifrac analysis), 16S rRNA Operational Taxonomic Units (OTUs) were defined at ≥ 99 % sequence homology using uclust^75^ and OTUs with less than 10 sequences were filtered. All reads were classified to the lowest possible taxonomic rank using QIIME^74^ and a reference dataset from the SILVA database. Biodiversity of the samples (alpha-diversity) were calculated with Chao1 and Shannon indexes. Similarities between samples (beta-diversity) were calculated by unweighted uniFrac^76^. The range of similarities is calculated between the values 0 and 1. PCoA representations of beta-diversity were performed using QIIME ^74^.

### TNBS-induced experimental colitis

For TNBS-induced acute colitis, mice were rectally challenged with 3 mg of Picrylsulfonic acid (TNBS, MW 293.17 Fluka). The weight curve was determined by weighing mice daily. At sacrifice colons were collected, their length was measured and divided in portions to be fixed in 10% formalin for histological analyses, snap-frozen for RNA extraction and for lamina propria mononuclear cells (LPMC) immunophenotyping.

### Metabolomic analysis

Metabolome extraction, purification and derivatization was carried by means of the MetaboPrep kit (Theoreo srl, Montecorvino Pugliano [SA], Italy) according to the manufacturer’s instruction.

Two µL samples of the derivatized solution were injected into the GC-MS system (GC-2010 Plus gas chromatograph coupled to a 2010 Plus single quadrupole mass spectrometer; Shimadzu Corp., Kyoto, Japan). Chromatographic separation was achieved with a 30 m 0.25 mm CP-Sil 8 CB fused silica capillary GC column with 1.00 µm film thickness from Agilent (Agilent, J&W Scientific, Folsom, CA, USA), with helium as carrier gas. The initial oven temperature of 100 °C was maintained for 1 min and then raised by 4 °C/min to 320 °C with a further 4 min of hold time. The gas flow was set to obtain a constant linear velocity of 39 cm/s and the split flow was set at 1:5. The mass spectrometer was operated in electron impact (70 eV) in full scan mode in the interval of 35-600 m/z with a scan velocity of 3333 amu/sec and a solvent cut time of 4.5 min. The complete GC program duration was 60 min. Untargeted metabolites were identified by comparing the mass spectrum of each peak with the NIST library collection (NIST, Gaithersburg, MD, USA). To identify metabolites identity, the linear index difference max tolerance was set at 10, while the minimum matching for the NIST library search was set at 85%. The chromatographic data for PLS-DA analysis were tabulated with one sample per row and one variable (metabolite) per column. According to MSI level 1 standard^77^, the VIP putative metabolites identity was confirmed by means of an independent analytical standard analysis. The normalization procedures consisted of data transformation and scaling. Data transformation was made by generalized log transformation and data scaling by autoscaling (mean-centered and divided by standard deviation of each variable).

### Metabolomics data analysis

Partial least square discriminant analysis (PLS-DA) ^78^ was performed on Internal Standard peak area ^79^ normalized chromatogram using R (Foundation for Statistical Computing, Vienna, Austria). Mean centering and unit variance scaling was applied for all analyses. Classes separation was archived by PLS-DA, which is a supervised method that uses multivariate regression techniques to extract, via linear combinations of original variables (X), the information that can predict class membership (Y). PLS regression was performed using the plsr function included in the R pls package^80^. Classification and cross-validation was performed using the corresponding wrapper function included in the caret package. A permutation test was performed to assess the significance of class discrimination. In each permutation, a PLS-DA model was built between the data (X) and the permuted class labels (Y) using the optimal number of components determined by cross validation for the model based on the original class assignment. Variable Importance in Projection (VIP) scores were calculated for each component. A VIP is a weighted sum of squares of the PLS loadings, taking into account the amount of explained Y-variation in each dimension.

To identify the most meaningful changes in two conditions, the volcano plot was used. This combines a measure of statistical significance from a statistical test (*p* value) with the magnitude of the change, enabling quick visual identification of those data points (metabolites) that display large magnitude changes that are also statistically significant. The volcano plots were constructed by plotting the negative log of the *p* value on the *y* axis. This results in data points with low p values (highly significant) appearing toward the top of the plot. The *x* axis was the log of the fold change between the two conditions. The log of the fold change is used so that changes in both directions appear equidistant from the center. Plotting points results in two regions of interest in the plot: those points that are found toward the top of the plot that are far to either the left- or right-hand sides. These represent values that display large magnitude fold changes (hence being left or right of center) as well as high statistical significance (hence being toward the top).

### In vitro T-cell activation assay

Colonic lamina propria and mesenteric lymph node leukocytes were collected from untreated mice. 2 × 10^6^ cells were plated and exposed to 0.05 mg of bacteria (wet weight) derived from faeces of untreated, DSS-treated or FMT-treated mice at the time of sacrifice. Gentamycin (50 µg/ml) and a cocktail of antibiotics (P/S) were added after 2 h of incubation. The cells were left in culture for 96 h. In some experiments, anti-MHCII blocking antibody (clone M5/114.15.2, TONBO) was added at a final concentration of 10 µg/ml. At the end of the experiment cells were analysed with flow cytometry. Their viability was checked with Zombie Yellow™ Fixable Viability Kit (Biolegend).

### Tissue ELISA of murine IL-10

Colonic tissues were homogenized in 300 µl RIPA Buffer (Cell Signaling Technology) supplemented with Phosphatase inhibitors (Sigma) and Protease inhibitors (Complete Ultra tablets, Roche). The samples were then incubated at 4 °C for 30 min under slow rotation and then centrifuged at 13,000 r.p.m. (16.2 × *g*) for 15 min at 4 °C. The supernatant was quantified at the NanoDrop with Bradford Assay (BioRad). mIIL-10 was measured on 6.25 µg of lysate using the ELISA assay (Purified anti-mouse IL-10 and Biotin anti-mouse IL-10, Biolegend) performed following manufacturer’s instructions.

### Faecal bacteria plating

One faecal pellet from each mouse was smashed, filtered with 100 µm nylon cell strainer and resuspended in 1 mL of sterile PBS. A concentration of 200 µL were then plated on Chocolate II Agar plates (BD) and grown at 37 °C under aerobic conditions overnight or in anaerobic conditions for 48 h.

### Quantification and statistical analysis

Statistical analysis was performed with GraphPad Prism 5 (GraphPad Software). Statistical significance was calculated using a Mann–Whitney test for comparison within two groups or Kruskal–Wallis test with Dunn’s multiple comparison correction within more than two groups. *P < 0.05, **P < 0.01, ***P < 0.001 were regarded as statistically significant. Outliers were detected with Grubb’s test. Non parametric distributions were represented as median+/− interquartile range.

## Electronic supplementary material

Supplementary Information

Reporting Summary

## Data Availability

16S rRNA raw data for Figs. 2a–f, 3a, b, 4d, f, 8b, e and Supplementary 4a, b, 5a, c, are available in SRA Online Repository associated to BioProject PRJNA494680.
