# Supplementary material for: Therapeutic faecal microbiota transplantation controls intestinal inflammation through IL10 secretion by immune cells
Source: Nat Commun. 2018 Dec 5;9:5184. doi: 10.1038/s41467-018-07359-8 (PMC6281577; doi:10.1038/s41467-018-07359-8)
Supplement: Supplementary file 1 — Supplementary Information [file 41467_2018_7359_MOESM1_ESM.pdf]

Therapeutic faecal microbiota transplantation controls intestinal inflammation through IL10 secretion by immune cells

Burrello et al.

Supplementary Figures

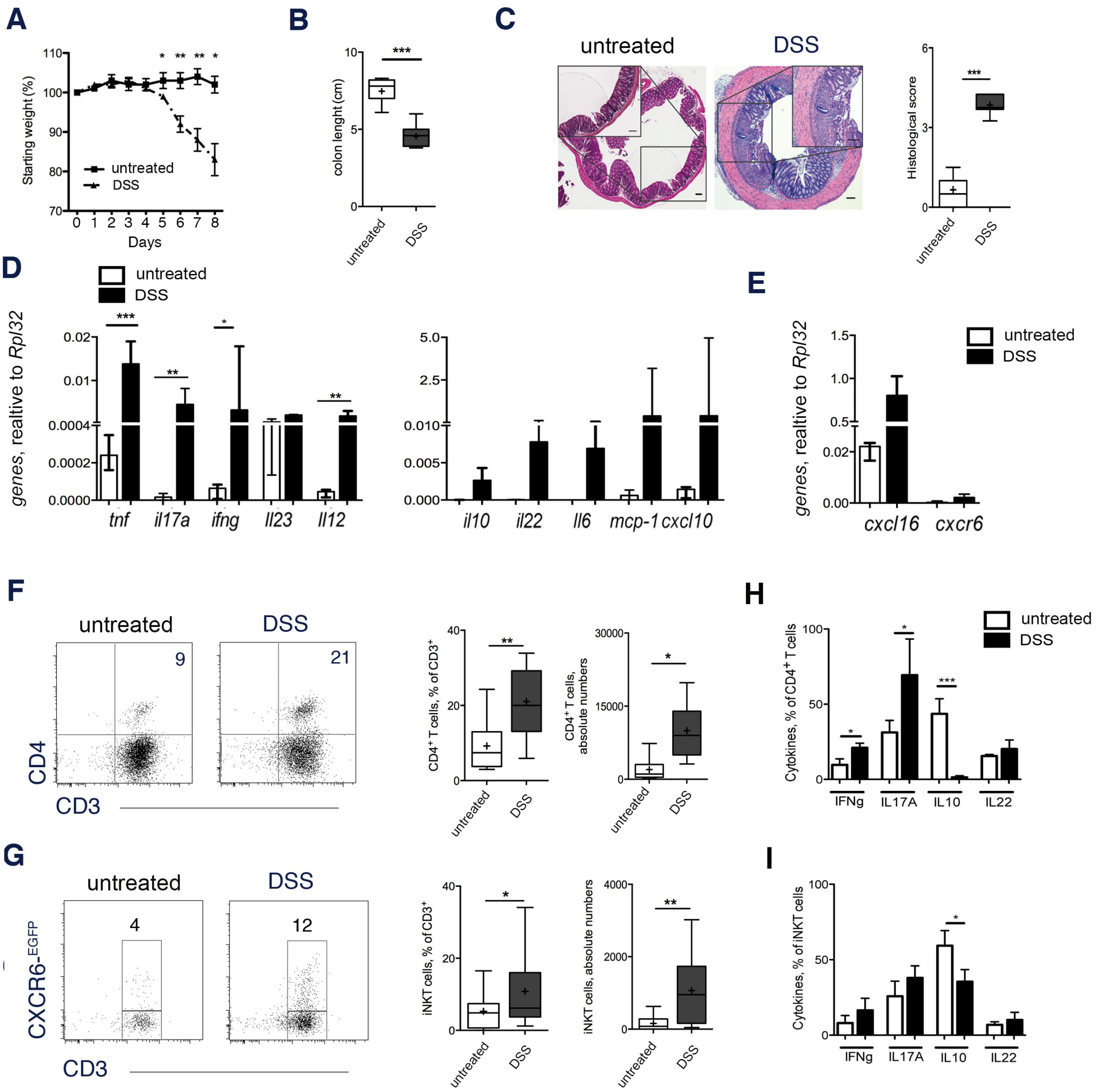

Supplementary Figure 1

Supplementary Figure1: *iNKT and CD4^{+} T cells exhibit a pro-inflammatory profile during acute intestinal inflammation in CXCR6 ^{EGFP} mice.* Acute experimental colitis was induced in CXCR6-$^{EGFP/+}$ mice by DSS administration in drinking water. (A) Weight loss, (B) colon length and (C) histological score were compared in mice treated with DSS (closed circles) or water (open circles). (D) Colonic expression levels of *tnf, il17a, ifng, il23, il12, il10, Il22, il6, mcp-1, cxcl10* in untreated (white bars)

and DSS-treated (black bars) mice by qPCR. (E) Colonic expression levels of *cxcl16 and cxcr6* in untreated (white bars) and DSS-treated (black bars) mice by qPCR (F,G) $CD4^+$ T cells (F, $CD45^+lin^-CD3^+EGFP^-CD4^+$) and iNKT cells (G, $CD45^+lin^-CD3^+$ $EGFP^+$) representative dot plots, frequency among $CD3^+$ T cells and absolute numbers in colon of DSS-treated (black bars) or controls (white bars). (H,I) Cytokine production by $CD4^+$ T cells (H) and iNKT cells (I) in DSS-treated and untreated mice. Controls n=12, DSS-treated n=21 from 5 independent experiments. Outliers were detected with Grubb's test. Significance was determined by using unpaired two-tailed Mann-Whitney test and is expressed as mean±SEM. $P < 0.05$ (*), $P < 0.01$ (**) $P < 0.001$ (***) were regarded as statistically significant.

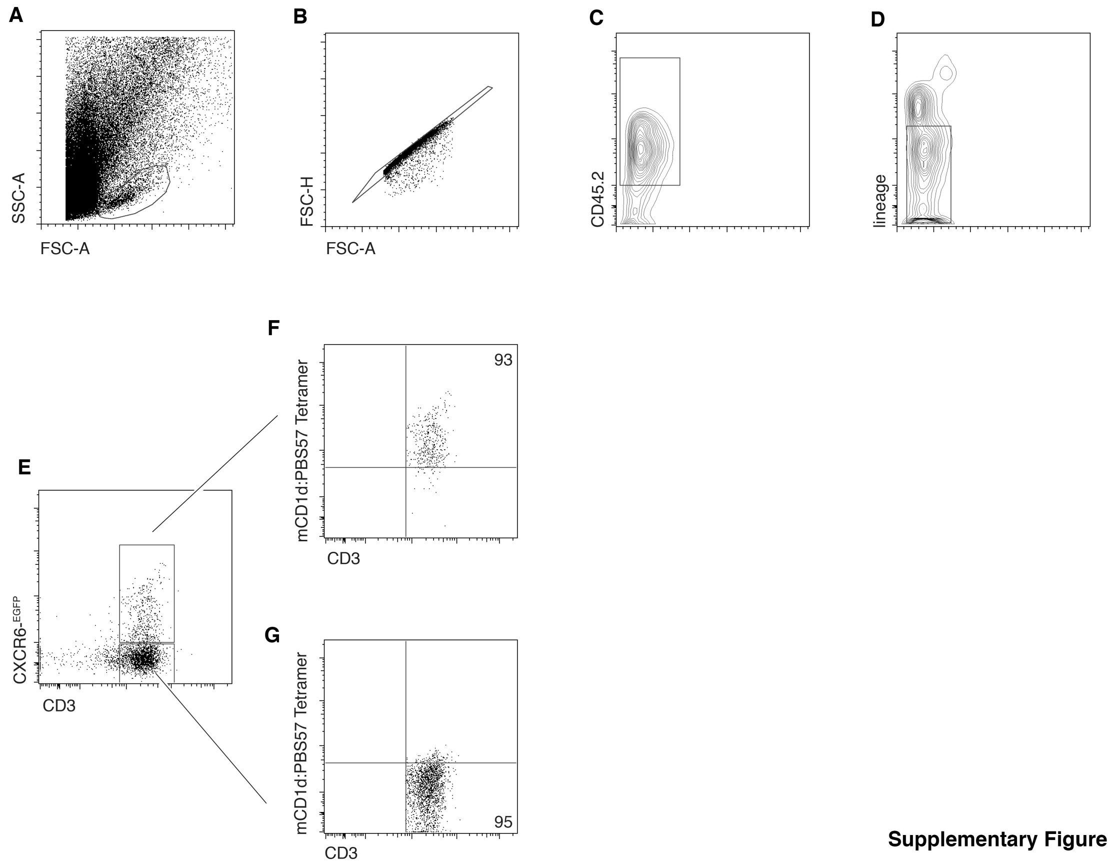

Supplementary Figure 2

Supplementary Figure 2: *Gating strategy to identify iNKT cells in CXCR6 $^{EGFP/+}$ mice.* (A) Forward and Side Scatter of colonic LPMC gate indicates living lymphocytes (B) Doublets-excluding gate (C) Epithelial cell-excluding gate based on CD45.2 expression. (D) Lineage (CD19, CD11c, CD11b)-excluding gate. (E) CD3 expression and CXCR6^{EGFP} (F) mCD1d:PBS57 Tet staining on gated CD3^{+} EGFP^{+} cells confirms that 93% of EGFP^{+} cells are iNKT cells. (G) mCD1d:PBS57 Tet staining on gated CD3^{+}EGFP^{-} cells.

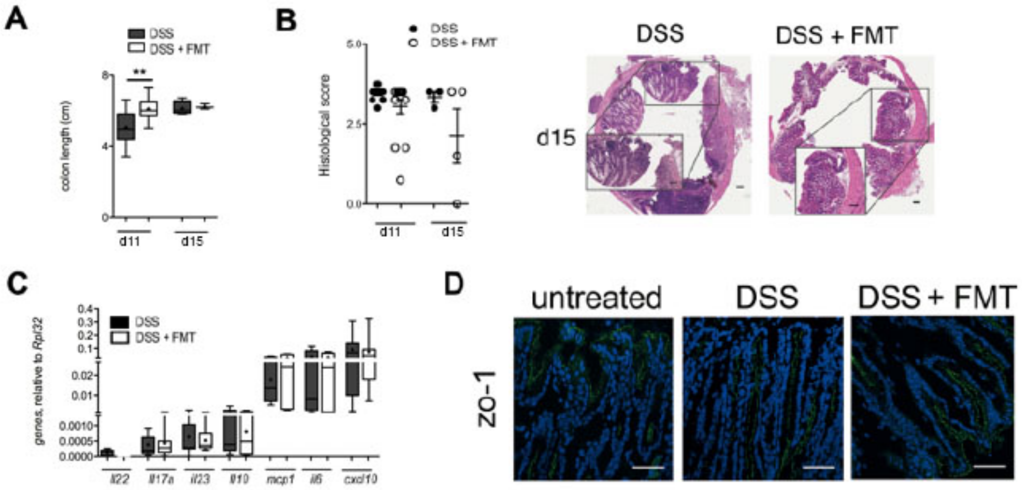

Supplementary Figure 3

Supplementary Figure 3: *FMT effects in acute and in recovery phase of DSS-induced intestinal inflammation*. (A,B) Colon length (A) and cumulative histological score (B) of DSS (closed circles) and FMT-treated (open circles) mice at day 11 or 15 after start of DSS administration. (C) Colonic expression levels of *il22, il17a, il23, il10, mcp-1, il6, cxcl10,* in DSS-treated mice (black bars) and FMT-treated mice (white bars). (D) zo-1 IF staining of intestinal mucosa of untreated (left), DSS treated (middle) and DSS+FMT treated (right) mice. Scalebar, 10 µm. Significance was determined using unpaired two-tailed Mann-Whitney test and expressed as mean± SEM. Outliers were detected with Grubb's test. P < 0.05 (*), P < 0.01 (**) were regarded as statistically significant

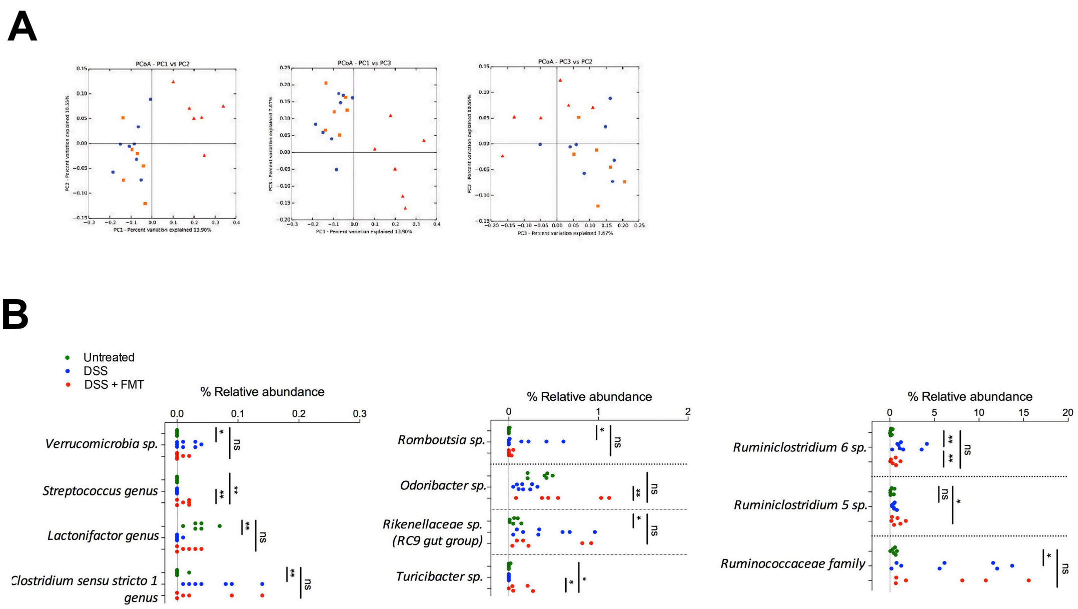

Supplementary Figure 4

Supplementary Figure 4 (A) Microbiome clustering based on unweighted Principal Coordinate Analysis (PCoA) UniFrac metrics of fecal gut microbiota derived from DSS treated (blue dots), DSS+FMT treated (orange dots) and untreated (red dots) mice. (B) Relative abundance of OTUs which differ between untreated (green dots), DSS (blue dots) and DSS+FMT (red dots) treated mice. Statistical significant difference was assessed through One way ANOVA with LSD post-hoc test * $p<0.05$, ** $p<0.01$.

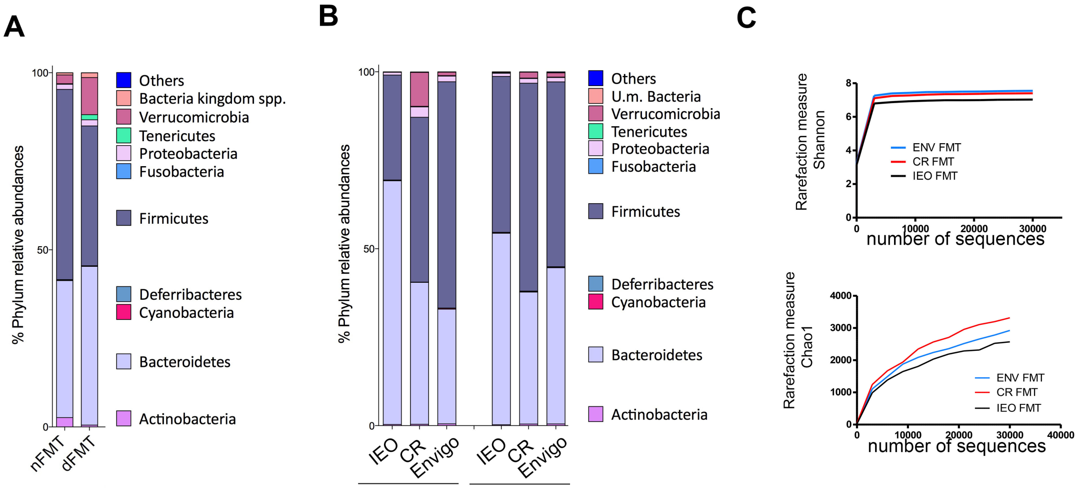

Supplementary Figure 5

Supplementary Figure 5: (A,B) Bar plots of the taxonomic composition showing relative abundances >1% of bacterial phyla from normobiotic and disbiotic (A) or IEO, CR and Envigo (B) FM donors. (C) Rarefaction curves showing microbial richness and evenness on the Shannon index (upper panel) and microbial richness based on the Chao1 index (lower panel).

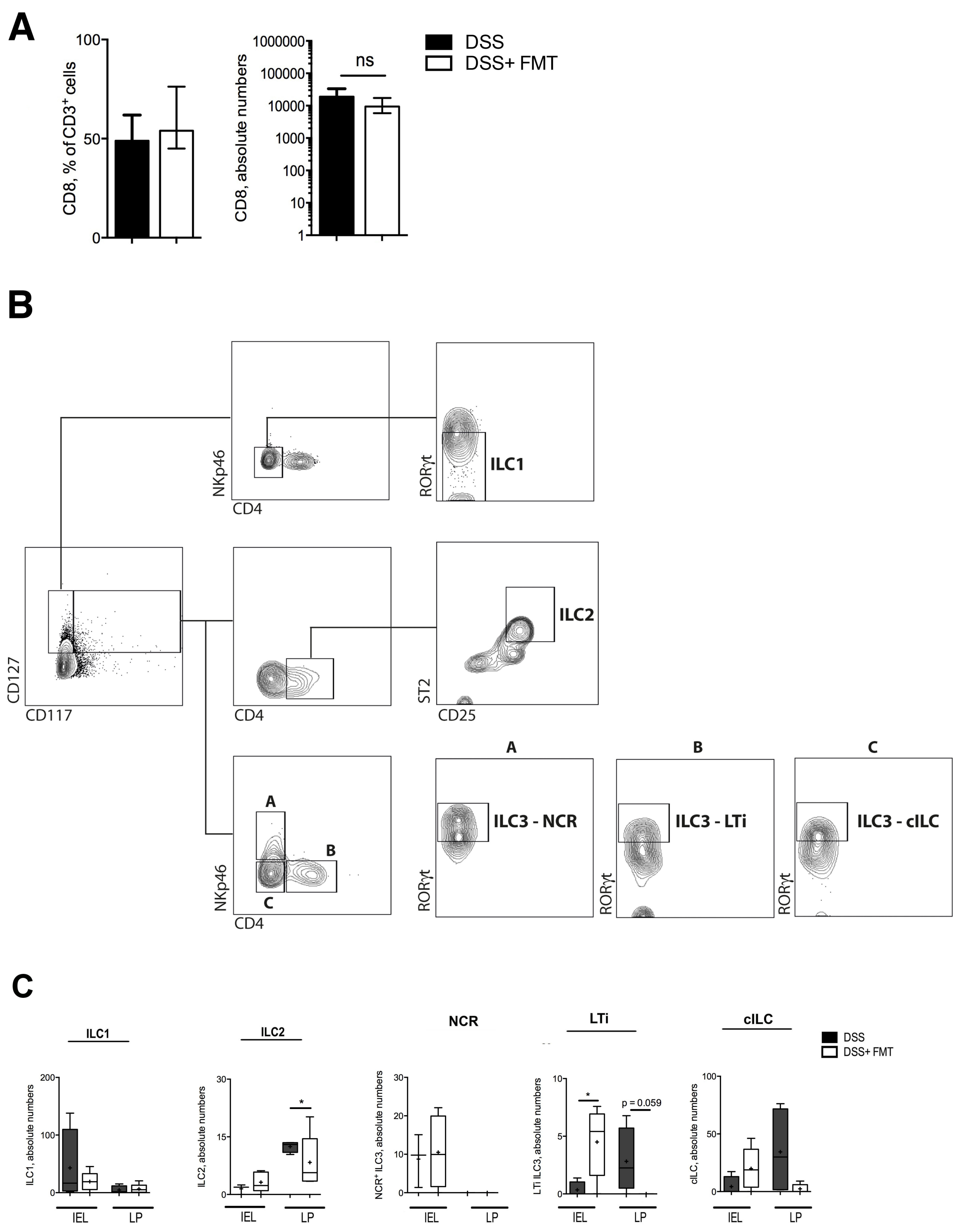

Supplementary Figure 6

Supplementary Figure 6: *Analysis of CD8^{+} T cells and innate lymphoid cells (ILC)*. (A) frequency and absolute numbers of colonic CD8+ T cells in DSS (black bars) and DSS+ FMT treated (white bars) mice. (B,C) Gating strategy to identify Lamina propria

(LP) and intra-epithelial (IEL) ILC1, ILC2, ILC3 (NCR, LTi, cILC) (B) and absolute numbers (C) of cells in DSS (black bars) and DSS+ FMT treated (white bars) mice.

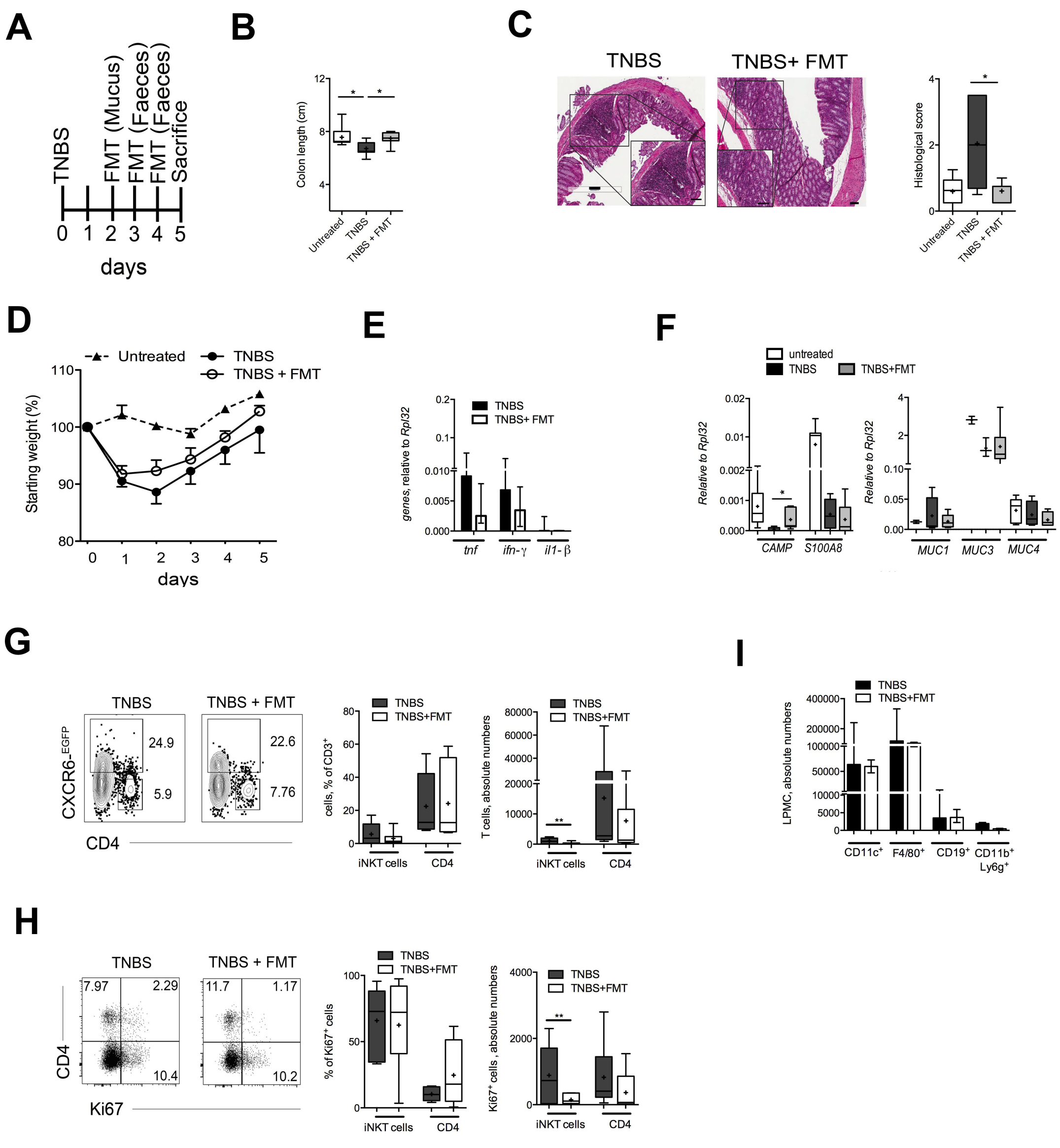

Supplementary Figure 7

Supplementary Figure 7: (A) *Therapeutic FMT ameliorates TNBS- induced colitis* (A) Schematic representation of FMT treatment during acute TNBS experimental colitis. (B-D) Colon length (B), H&E staining (scalebar 100μm) and cumulative histological score (C) and weight loss (D) of untreated (closed triangles), colitic (closed circles), or colitic mice treated with FMT (open circles). (E) Colonic expression levels of *tnf, ifnγ* and *il1β* in colitic (black bars) and FMT-treated (white bars) mice. (F) Colonic expression levels of *camp*, *S100A8 (left panels)* and *muc1,*

muc3, muc4 (right panels) in untreated (white bars), TNBS treated (black bars) or TNBS+FMT treated (gray bars) mice. (G) Representative dot plots (left panels), frequencies and absolute numbers (right panels) of colonic $CD4^+$ T cells and iNKT cells in TNBS-treated (black bars) and FMT-treated (white bars) mice 5 days post TNBS administration (H) Representative dot plots, (left panels), frequencies and absolute numbers (right panels) of Ki67-expressing colonic $CD4^+$ T cells and iNKT cells in TNBS-treated (black bars) and FMT-treated (white bars) mice. (I) Lamina propria (LP) and intra-epithelial (IEL) ILC1, ILC2, ILC3 (NCR, LTi, cILC) absolute numbers from acute TNBS (black bars) and FMT-treated mice (white bars). (J) Absolute numbers of colonic DC, macrophages, B cells, neutrophils from acute TNBS (black bars) and FMT-treated mice (white bars).

Total TNBS-treated n=16, TNBS+FMT n=21 mice from 2 independent experiments. Outliers were detected with Grubb's test. Significance was determined using unpaired two-tailed Mann-Whitney test and expressed as median±SD. $P < 0.05$ (*), $P < 0.01$ (**) $P < 0.001$ (***) were regarded as statistically significant.

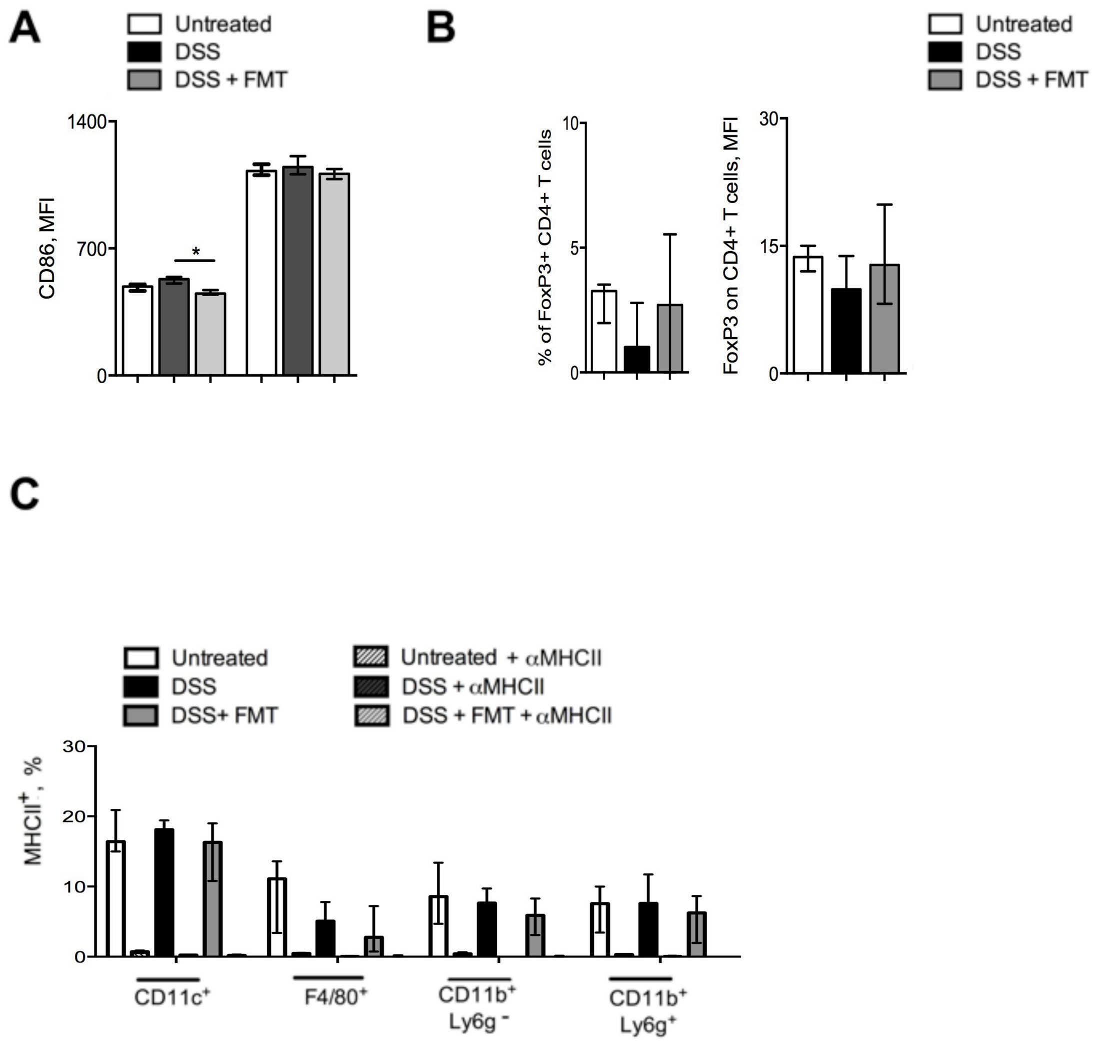

Supplementary Figure 8

Supplementary Figure 8: *In vitro antigen presentation of bacterial antigens***.** (A) Frequency of CD86+ dendritic cells ($CD45.2^{+}CD3^{-}CD11c^{+}$) and macrophages ($CD45.2^{+}CD3^{-}F4/80^{+}$), exposed in vitro to faeces of untreated (white bars), DSS-treated (dark grey bars), DSS+ FMT-treated (light grey bars) mice in the presence (striped bars) or absence (filled bars) of neutralizing anti-MHC-II antibody. (B) Frequency of Foxp3+ Treg cells (left panel) and MFI of Foxp3 on intestinal $CD4^{+}$ T cells after in vitro exposure to faeces from untreated (white bars), DSS-treated (black bars), DSS+FMT-treated (grey bars) (C) Frequency of MHCII+ splenic dendritic cells ($CD45.2^{+}CD3^{-}CD11c^{+}$), macrophages ($CD45.2^{+}CD3^{-}F4/80^{+}$),

monocytes ($CD45.2^+CD3^-CD11b^+Ly6g^-F4/80^-$), neutrophils ($CD45.2^+CD3^-Ly6g^+CD11b^+$) exposed in vitro to faeces of untreated (white bars), DSS-treated (black bars), DSS+ FMT-treated (grey bars) mice in the presence (striped bars) or absence (filled bars) of neutralizing anti-MHC-II antibody.

Significance was determined using unpaired two-tailed Mann-Whitney test and expressed as mean±SEM. Outliers were detected with Grubb's test. $P < 0.05$ (*),$P < 0.001$ (***) were regarded as statistically significant.

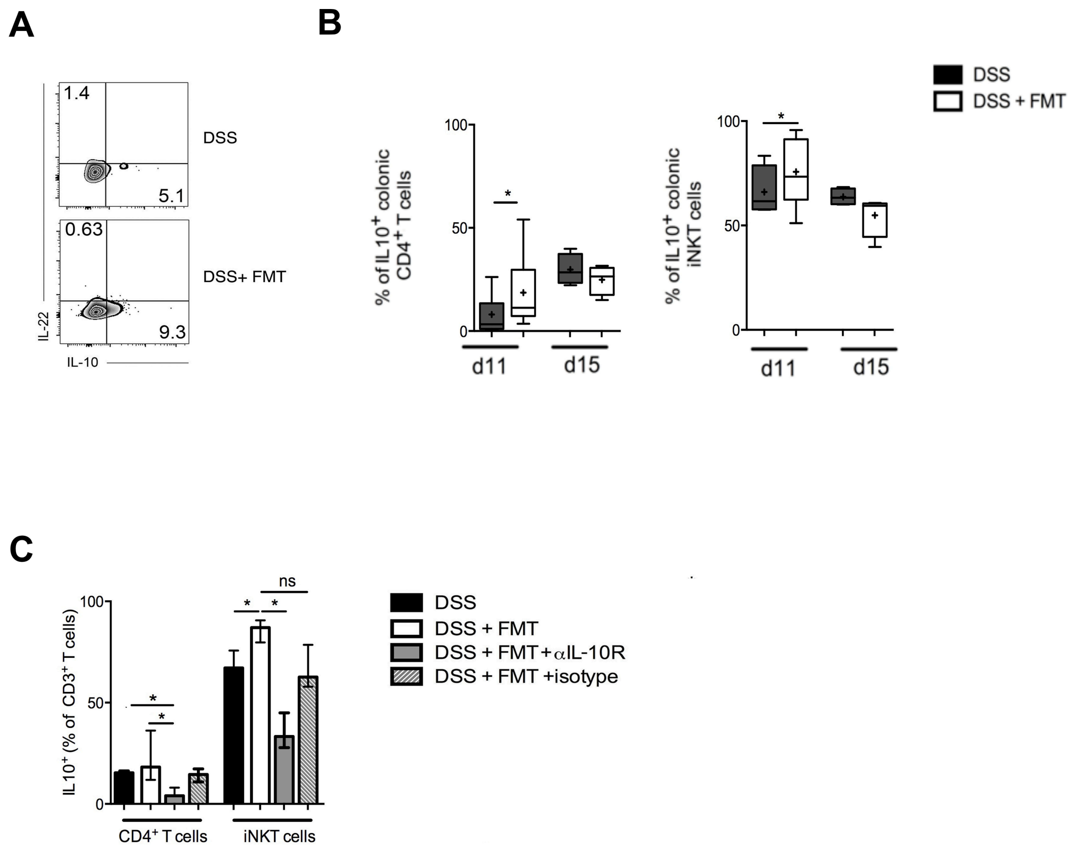

Supplementary Figure 9

Supplementary Figure 9: *IL10 induction upon FMT* (A,B) Representative dot plots (A) and frequencies (B) of cytokines secreted by colonic CD4$^+$ T (left panels) and iNKT cells (right panels) in DSS-treated (black bars) and FMT-treated (white bars) mice 11 days and 15 days after starting DSS administration. (C) Colonic expression of *il1β* in DSS treated mice (black bars), DSS+FMT treated (white bars), DSS+FMT+αIL-10R treated (grey bars) and DSS+FMT+αIL-10R isotype antibody treated mice (grey striped bars). (D) IL-10 production by CD4$^+$ and iNKT cells in DSS treated mice (black bars), DSS+ FMT treated (white bars), DSS+FMT+αIL-10R treated (grey bars) and DSS+FMT+αIL-10R isotype antibody treated mice (grey striped bars)

Significance was determined using unpaired two-tailed Mann-Whitney test and expressed as mean±SEM. Outliers were detected with Grubb's test. $P < 0.05$ (*) were regarded as statistically significant.

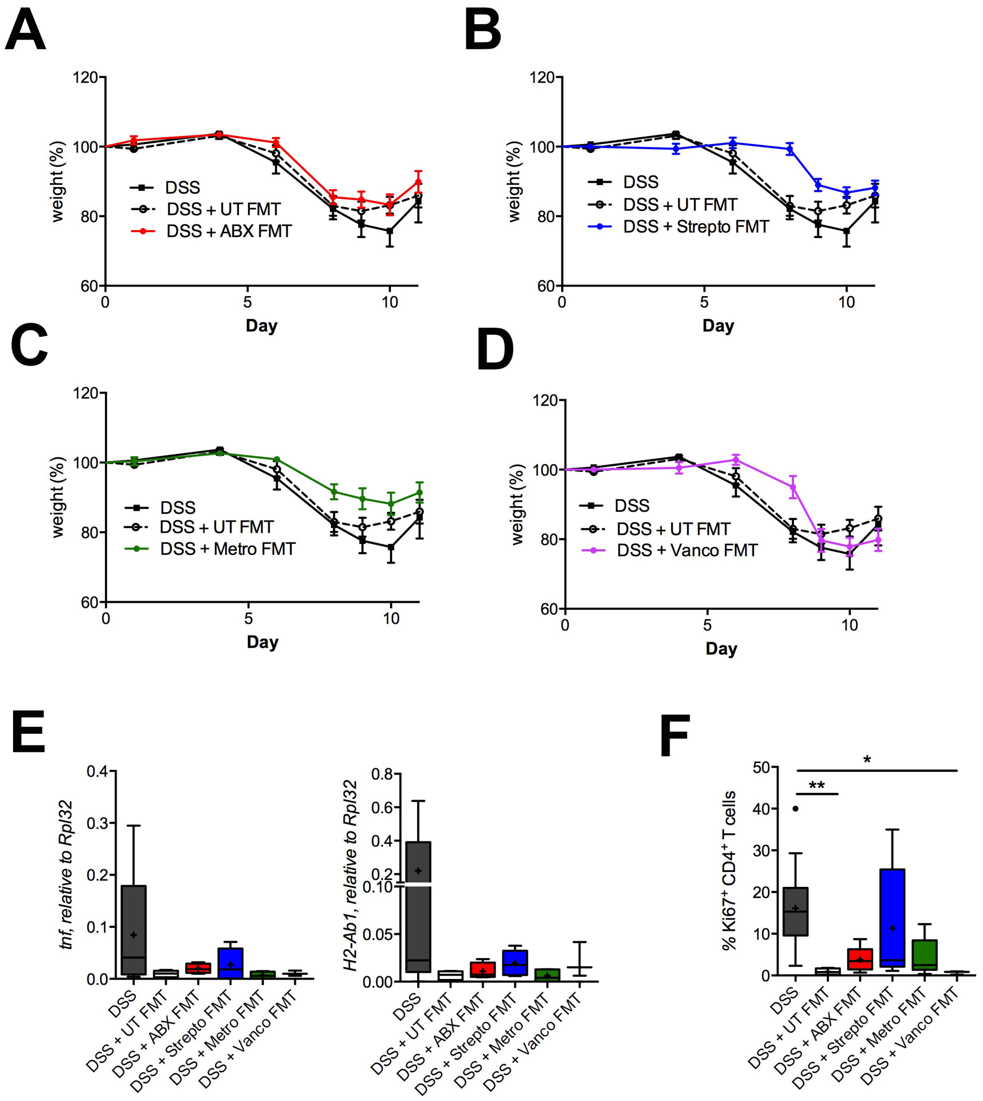

Supplementary Figure 10

Supplementary Figure 10. (A-D) Weight curves of DSS-treated (black squares), DSS+ UT FMT-treated (black circles) and (A) DSS+ ABX FMT (red curves) (B), DSS+ Strepto FMT (blue curves), (C), DSS+ Metro FMT (green curves), (D), DSS+ Vanco FMT (violet curves). (E) tnf (left panel) and H2-ab1 (right panel) colonic expression in DSS- treated (black bars), DSS+ untreated FMT (white bars), DSS +

ABX FMT (red bar), DSS + Streptomycin FMT (blue bar), DSS+ Metronidazole FMT (green bar) and Vancomycin (Violet bar)-treated mice. (F) Ki67 expression in colonic CD4+ T cells in DSS- treated (black bars), DSS+ untreated FMT (white bars), DSS + ABX FMT (red bar), DSS + Streptomycin FMT (blue bar), DSS+ Metronidazole FMT (green bar) and Vancomycin (Violet bar)-treated mice.

Supplementary Tables

Supplementary Table 1 Scoring scheme for evaluation of intestinal inflammation

	Criterion	Definition	Score value
**Inflammatory cell infiltrate**	Severity (leukocyte density of lamina propria area infiltrated in evaluated hpf)	No infiltrate	0
		Minimal acute (<10%)	0.25
		Mild chronic (10-25%, scattered neutrophils)	0.5
		Moderate chronic (26-50%)	0.75
		Marked (>51%, dense infiltrate)	1
	Extent (expansion of leukocyte infiltration)	Mucosal	0.5
		Mucosal and submucosal	0.75
**Epithelial changes**	Hyperplasia (increase in epithelial cell numbers in longitudinal crypts, visible as crypt elongation)	No hyperplasia	0
		Minimal (<25%)	0.25
		Mild (26-35%)	0.5
		Moderate (36-50%, mitoses in the upper third of the crypt epithelium)	0.75
		Marked (>51%, mitoses in crypt epithelium distant from crypt base)	1
	Goblet cell loss (reduction of goblet cell numbers relative to baseline goblet cell numbers per crypt)	No loss	0
		Minimal (<25%)	0.25
		Mild (26-35%)	0.5
		Moderate (36-50%)	0.75
		Marked (>51%)	1
**Mucosal architecture**	Ulceration (epithelial defect reaching beyond muscolaris mucosae)	No ulcers	0
		Ulcers	0.25
	Granulation tissue (connective tissue repair with new capillaries, surrounded by infiltrating cells, hypertrophied areas)	No granulation tissue	0
		Granulation tissue	0.25
	Mucosal thickness and crypt	No thickening	0

	depth		
		Thickening	0.5
	Glandular rarefaction	No rarefaction	0
		Rarefaction	0.5
	Dysplasia	No dysplasia	0
		Dysplasia	0.5
		**MAX SCORE**	**6**

Supplementary Table 2 Primer sequences

Primer	Product			Vendor
IL17a	Qiagen (QuantiTect)			QT00103278
IFNg	Qiagen (QuantiTect)			QT01038821
IL10	Qiagen (QuantiTect)			QT00106169
IL23	Qiagen (QuantiTect)			QT01663613
IL22	Qiagen (QuantiTect)			QT00128324
Rpl32	Qiagen (QuantiTect)			QT00131992
Tjp1	Qiagen (QuantiTect)			QT00493899
Camp1	Qiagen (QuantiTect)			QT00241003
S100A8	Qiagen (QuantiTect)			QT00250264
IL1b	Qiagen (QuantiTect)			QT010483555
**Primer**	**Forward**	**Reverse**	**bp**	**Vendor**
CXCL16	AGCGCAAAGAGTGTGGA	GGTTGGGTGTGCTCT	193	SIGMA
CXCR6	CCTTTTTGGGCCTATGCA	ATGCCTCGAAGAGTT	71	SIGMA
MCP-1	CAAGATGATCCCAATGA	GGTTCCGATCCAGGT	161	SIGMA
CXCL10	CGCTGCAACTGCCATCCA	CCGGATTCAGACATC	148	SIGMA
TNF	TCTTCTCATTCCTGCTTG	CACTTGGTGGTTTGCT	200	SIGMA
IL6	CTCTGGGAAATCGTGGA	GCAAGTGCATCATCG	77	SIGMA
IL12	CCTGCTGAAGACCACAG	AGCTCCCTCTTGTTGT	200	SIGMA
Muc1	TACCCTACCTACCACACTCACG	CTGCTACTGCCATTACCTGC	95	SIGMA
Muc2	GTGTGGGACCTGACAATGTG	TTGCCACCAGAACATTTCTTT	124	SIGMA
Muc3	CTTCCAGCCTTCCCTAAACC	TCCACAGATCCATGCAAAAC	119	SIGMA
Muc4	GAGAGTTCCCTGGCTGTGTC	GGACATGGGTGTCTGTGTTG	101	SIGMA

Supplementary Table 3 FACS antibodies and dyes

Marker	Reactivity	Clone	Vendor	Cat. no	Dilution
mCD1d:PBS57 Tet	Mouse		Gift from NIH Tet facility		0.1µL/10^6 cells
CD45.2	Mouse	104	Biolegend	109838	1:200
CD3	Mouse	17A2	TONBO	35-0032-u100	1:200
CD8α	Mouse	53-6.7	Biolegend	100741	1:200
CD4	Mouse	RM4-5	BD	565650	1:200
CD11c	Mouse	N418	TONBO	25-0114-u025	1:200
CD19	Mouse	1D3	TONBO	75-0193-U100	1:200

CD11b	Mouse	M1/70	TONBO	25-0112-U100	1:200
F4/80	Mouse	BM8.1	TONBO	50-4801-U025	1:200
Ly6g	Mouse	1A8	TONBO	20-1276-U025	1:200
Ly6c	Mouse	HK14	Biolegend	128012	1:200
MHC-II	Mouse	M5/114.15.2	Biolegend	107641	1:200
Ki67	Mouse	16A8	Biolegend	652422	1:100
CD69	Mouse	H1.2F3	Biolegend	104521	1:200
Ter119	Mouse	TER-119	BD	553673	1:200
TCR α/β	Mouse	H57.597	BD	562839	1:200
TCR γ/δ	Mouse	UC713B5	BD	561996	1:200
CD90	Mouse	53-2.1	BD	561974	1:200
CD117	Mouse	2B8	BD	553355	1:200
CD127	Mouse	A7R34	Biolegend	135039	1:200
Nkp46	Mouse	29A1.4	BD	741029	1:200
RorγT	Mouse	AFKJS9	eBioscience	17-6988-82	1:100
ST-2	Mouse	DIH9	Biolegend	145303	1:200
CD25	Mouse	PC61	BD	557192	1:200
FoxP3	Mouse	FJK-16s	eBioscience	12-5773-80	1:100
IFNγ	Mouse	XMG1.2	BD	564336	1:100
IL10	Mouse	JES5-16E3	BD	563276	1:100
IL22	Mouse	Poly5164	Biolegend	516411	1:100
IL17A	Mouse	TC11-18H10.1	Biolegend	506920	1:100
TNF	Mouse	MP6-XT22	Biolegend	506327	1:100
IL13	Mouse	eBio13A	eBioscience	25-7133-82	1:100
Zombie Dye	Mouse	423104	Biolegend	423104	1μL/10^6 cells

Supplementary Table 4 Number of mice for each experimental group

		Experimental group													
Figure	Readout	UT	DSS	DSS + nFMT	DSS + dFMT	DSS + IEO FMT	DSS + Envigo FMT	DSS + CR FMT	DSS + aIL10R	DSS + FMT + aIL10R	DSS + FMT + iso	DSS + Abx FMT	DSS + Strepto FMT	DSS + Metro FMT	DSS + Vanco FMT
Figure 1	weight curve	10	10	10	-	-	-	-	-	-	-	-	-	-	-
	colon length	12	12	14	-	-	-	-	-	-	-	-	-	-	-
	histology	8	15	19	-	-	-	-	-	-	-	-	-	-	-

	tissue gene expression	5	6	7	-	-	-	-	-	-	-	-	-	-	-
Figure 2	Metagenomic analyses	6	8	6	-	-	-	-	-	-	-	-	-	-	-
	Metabolomic analyses	6	11	13	-	-	-	-	-	-	-	-	-	-	-
Figure 3+4	Metagenomic analyses	6	8	7	7	9	10	9	-	-	-	-	-	-	-
	Metabolomic analyses	-	5	10	11	6	5	5	-	-	-	-	-	-	-
	colon length	-	7	13	7	10	10	10	-	-	-	-	-	-	-
	tissue gene expression	-	7	13	7	10	10	10	-	-	-	-	-	-	-
Figure 5	FACS (T cells)		12	12	-	-	-	-	-	-	-	-	-	-	-
	FACS (APC)		16	16	-	-	-	-	-	-	-	-	-	-	-
Figure 6		5	7	9	-	-	-	-	-	-	-	-	-	-	-
Figure 7	IL-10, protein lysate	-	15	26	-	-	-	-	-	-	-	-	-	-	-
	IL-10, FACS	-	14	17	-	-	-	-	-	-	-	-	-	-	-
	anti IL-10R blocking antibody	-	4	4	-	-	-	-	5	5	3	-	-	-	-
Figure 8	colon length	-	4	5	-	-	-	-	-	-	-	6	6	6	3
	tissue gene expression	-	4	5	-	-	-	-	-	-	-	6	6	6	3
	Metagenomic analysis	-	4	5	-	-	-	-	-	-	-	5	6	6	3
	FACS	-	4	5	-	-	-	-	-	-	-	6	6	6	6
